# Tandem inactivation of inositol pyrophosphatases Asp1, Siw14, and Aps1 illuminates functional redundancies in inositol pyrophosphate catabolism in fission yeast

**DOI:** 10.1128/mbio.00389-25

**Published:** 2025-04-16

**Authors:** Beate Schwer, Isabel Prucker, Ana M. Sanchez, Jill Babor, Henning J. Jessen, Stewart Shuman

**Affiliations:** 1 Department of Microbiology and Immunology, Weill Cornell Medical College 12295 , New York, New York, USA; 2 Institute of Organic Chemistry, University of Freiburg 9174, Freiburg, Baden-Württemberg, Germany; 3 Molecular Biology Program, Sloan Kettering Institute 332353 , New York, New York, USA; 4 Gerstner Sloan Kettering Graduate School of Biomedical Sciences 132190 , New York, New York, USA; 5 CIBSS-Centre for Integrative Biological Signaling Studies, University of Freiburg 9174, Freiburg, Baden-Württemberg, Germany; Harvard Medical School, Boston, Massachusetts, USA

**Keywords:** *Schizosaccharomyces pombe*, inositol pyrophosphates, pyrophosphatases

## Abstract

Inositol pyrophosphates 5-IP_7_, 1-IP_7_, and 1,5-IP_8_ are eukaryal signaling molecules that influence cell physiology, especially phosphate homeostasis. In fission yeast, 1,5-IP_8_ and 1-IP_7_ impact gene expression by acting as agonists of RNA 3'-processing and transcription termination. 1,5-IP_8_ is synthesized by position-specific kinases Kcs1 and Asp1 that convert IP_6_ to 5-IP_7_ and 5-IP_7_ to 1,5-IP_8_, respectively. Inositol pyrophosphatase enzymes Asp1 (a histidine acid phosphatase), Siw14 (a cysteinyl phosphatase), and Aps1 (a Nudix hydrolase) are agents of inositol pyrophosphate catabolism in fission yeast. Whereas Asp1, Siw14, and Aps1 are individually inessential, double pyrophosphatase mutants *asp1-H397A aps1*∆ and *siw14*∆ *aps1*∆ display severe growth defects caused by overzealous 3'-processing/termination. By applying CE-ESI-MS to profile the inositol pyrophosphate content of fission yeast mutants in which inositol pyrophosphate toxicity is genetically suppressed, we elucidated the functional redundancies of the Asp1, Siw14, and Aps1 pyrophosphatases. Asp1, which exclusively cleaves the 1-β-phosphate, and Aps1, which prefers to cleave the 1-β-phosphate, play essential overlapping roles in guarding against the accumulation of toxic levels of 1-IP_7_. Aps1 and Siw14 together catabolize the inositol-5-pyrophosphates, and their simultaneous inactivation results in overaccumulation of 5-IP_7_. Cells lacking all three pyrophosphatases amass high levels of 1,5-IP_8_ and 1-IP_7_, with concomitant depletion of IP_6_. A genetic screen identified three missense mutations in the catalytic domain of Kcs1 kinase that suppressed inositol-1-pyrophosphate toxicosis. The screen also implicated the 3'-processing factor Swd22, the inositol pyrophosphate sensor Spx1, and the nuclear poly(A)-binding protein Nab2 as mediators of inositol-1-pyrophosphate toxicity.

**IMPORTANCE:**

Inositol pyrophosphates are key effectors of eukaryal cellular phosphate homeostasis. They are synthesized by kinases that add a β-phosphate to the 5- or 1-phosphate groups of IP_6_ and catabolized by three classes of pyrophosphatases that hydrolyze the β-phosphates of 5-IP_7_, 1-IP_7_, or 1,5-IP_8_. Whereas the fission yeast inositol pyrophosphatases—Asp1 (histidine acid phosphatase), Siw14 (cysteinyl phosphatase), and Aps1 (Nudix hydrolase)—are inessential for growth, Asp1/Aps1 and Aps1/Siw14 double mutations and Asp1/Siw14/Aps1 triple mutations elicit severe or lethal growth defects. By profiling the inositol pyrophosphate content of pyrophosphatase mutants in which this toxicity is genetically suppressed, we reveal the functional redundancies of the Asp1, Siw14, and Aps1 pyrophosphatases. Their synergies are manifested as excess accumulation of 1-IP_7_ upon dual inactivation of Asp1 and Aps1 or an excess of 5-IP_7_ in *aps1*∆ *siw14*∆ cells. In the absence of all three pyrophosphatases, cells accrue high levels of 1,5-IP_8_ and 1-IP_7_ while IP_6_ declines.

## INTRODUCTION

**Fig 1:**
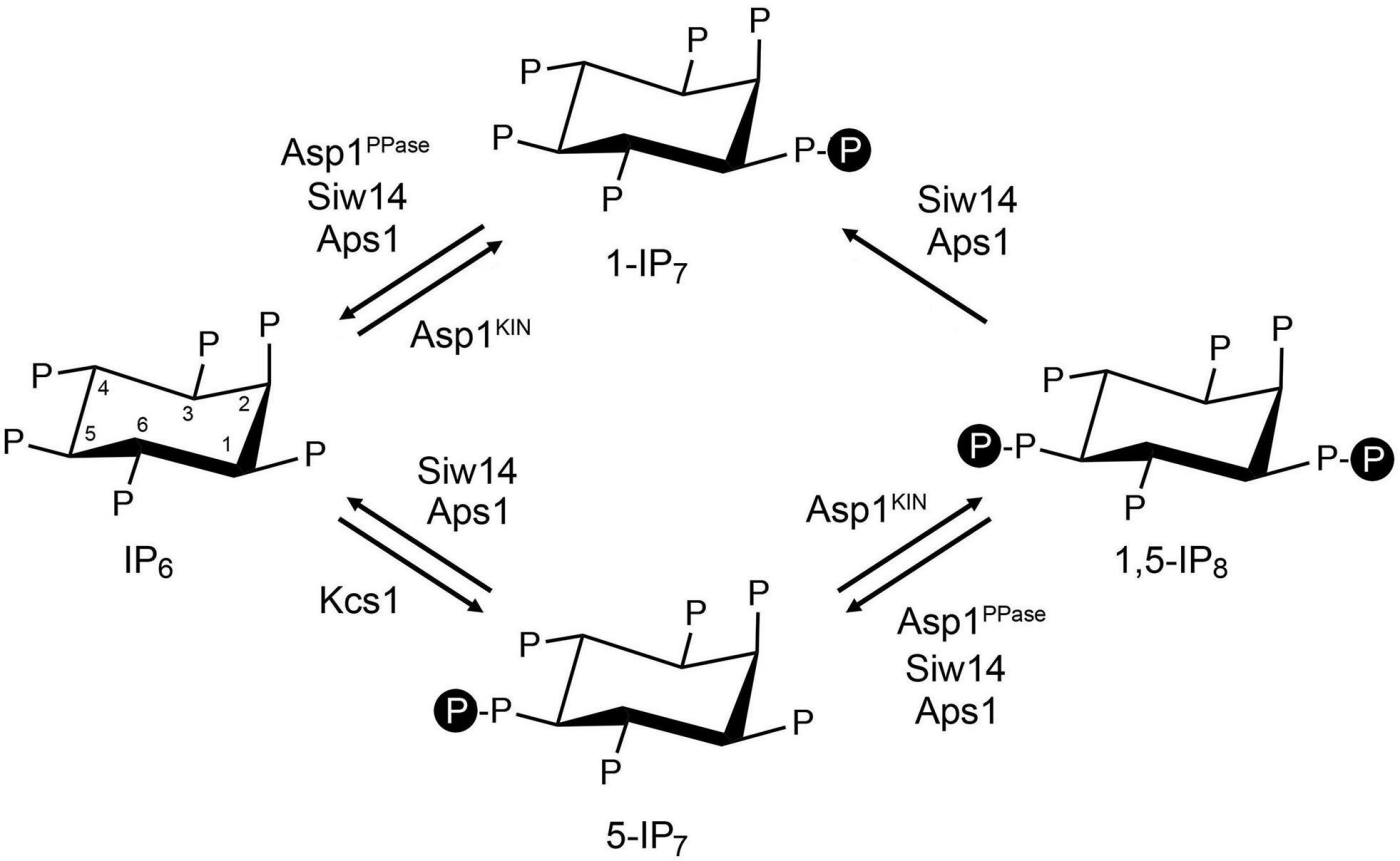
Inositol pyrophosphate metabolism in fission yeast. The chemical structures of IP_6_, 5-IP_7_, 1,5-IP_8_, and 1-IP_7_ are shown, with “P” denoting phosphate. The positions of the myo-inositol ring are indicated for IP_6_. The fission yeast enzymes that add (Kcs1 and Asp1-kinase) or remove (Siw14, Asp1-pyrophosphatase, Aps1) β-phosphate groups are indicated.

Inositol pyrophosphates IP_7_ and IP_8_ are eukaryal signaling molecules that influence cellular phosphate homeostasis (1). The isomers 5-IP_7_ and 1-IP_7_ differ as to whether the pyrophosphate moiety is at the 1 or 5 position of the inositol ring (Fig. 1). 1,5-IP_8_ is pyrophosphorylated at both positions. 1,5-IP_8_ is synthesized by the sequential action of position-specific kinases Kcs1/IP6K, which converts IP_6_ to 5-IP_7_, and Asp1/Vip1/VIH/PPIP5K (in fission yeast, budding yeast, plants, and humans), which converts 5-IP_7_ to 1,5-IP_8_ (1–8) (Fig. 1). Whereas the Asp1 kinase is also capable of converting IP_6_ to 1-IP_7_ *in vitro* (5), as are the Vip1/VIH/PPIP5K kinases (2, 4, 6), it is thought that the predominant route to 1-IP_7_ *in vivo* is via catabolism of 1,5-IP_8_ by inositol pyrophosphatase enzymes (8) (Fig. 1).

Asp1 and its orthologs Vip1/VIH/PPIP5K are bifunctional enzymes composed of an N-terminal domain that synthesizes 1,5-IP_8_ and a C-terminal pyrophosphatase domain, of the histidine acid phosphatase enzyme family, that hydrolyzes 1,5-IP_8_ back to 5-IP_7_ (2–8). The Asp1 pyrophosphatase can also convert 1-IP_7_ to IP_6_ *in vitro* (5), but the physiological relevance of this reaction is unclear. Two other classes of pyrophosphatases—Siw14 (9–12) and Aps1/Ddp1/DIPP (from fission yeast, budding yeast, and humans) (13–21)—are implicated in the catabolism of inositol pyrophosphates. Siw14 belongs to the cysteinyl-phosphatase family of metal-independent phosphohydrolases that catalyze phosphoryl transfer to water via a covalent enzyme-(cysteinyl-S)-phosphate intermediate. Aps1/Ddp1/DIPP belongs to the Nudix family of metal-dependent pyrophosphohydrolases. Fission yeast Siw14 is adept at converting 5-IP_7_, 1-IP_7_, and 1,5-IP_8_ to IP_6_, without significant positional bias as to the β-phosphate hydrolyzed *in vitro* (12) (Fig. 1). Fission yeast Aps1 cleaves the β-phosphate moieties from 5-IP_7_, 1-IP_7_, and 1,5-IP_8_ to yield IP_6_ as an end-product (21). Aps1 displays a twofold preference *in vitro* for hydrolysis of 1-IP_7_ versus 5-IP_7_ (21).

The fission yeast *asp1*^+^, *siw14*^+^, and *aps1*^+^ genes are individually inessential for vegetative growth. The availability of null, kinase-dead, and pyrophosphatase-dead *asp1* mutants has provided insights into the metabolism of inositol pyrophosphates. Monitoring the intracellular pools of IP_6_ and inositol pyrophosphates, initially by metabolic labeling with ^3^H-inositol and ion exchange chromatography (3, 4), or more recently via sensitive capillary electrophoresis electrospray ionization mass spectrometry (CE-ESI-MS) methods (21), has affirmed that (i) inactivation of the Asp1 kinase completely erases the pool of 1,5-IP_8_ and 1-IP_7_ and elicits a compensatory increase (by ~6-fold) in the level of 5-IP_7_; (ii) inactivation of the Asp1 pyrophosphatase results in a modest (~2-fold) increase in the levels of 1,5-IP_8_ and 1-IP_7_, without affecting the level of 5-IP_7_, consistent with its known specificity for hydrolysis of the 1-β-phosphate. Analysis of null mutants of *siw14* and *aps1* revealed that (i) *siw14*∆ elicits a slight increase in the levels of 1,5-IP_8_ (by 70%) and 5-IP_7_ (by 60%) without affecting 1-IP_7_; and (ii) *aps1*∆ cells have modestly increased levels of 1-IP_7_ (twofold), 5-IP_7_ (70%), and 1,5-IP_8_ (50%) (21). The relatively small impact of ablating the Asp1, Siw14, and Aps1 pyrophosphatase activities on inositol pyrophosphate levels *in vivo* suggests that there might be functional redundancy in their catabolism of fission yeast inositol pyrophosphates.

Indeed, the available genetic analyses underscore that this is the case. Simultaneous inactivation of the Asp1 pyrophosphatase (by active site mutation H397A) and deletion of the Aps1 pyrophosphatase is synthetically lethal (22), i.e., no viable *asp1-H397A aps1*∆ progeny were recovered on YES medium after crossing the respective single mutants. This result was taken to signify that too much 1,5-IP_8_ or 1-IP_7_ is toxic to fission yeast (22). Simultaneous deletion of Aps1 and Siw14 was also found to be synthetically lethal by the same criterion that a *siw14*∆ *aps1*∆ double mutant haploid could not be recovered on the YES medium after crossing the *siw14*∆ and *aps1*∆ single mutants (12). By contrast, an *asp1-H397A siw14*∆ double mutant grew as well as wild-type on YES medium at all temperatures (12).

A more sensitive gauge of the effects of inositol pyrophosphatase mutations on fission yeast physiology is their impact on the expression of the three genes that comprise a phosphate acquisition (*PHO*) regulon (23). The *PHO* genes *pho1* (cell surface acid phosphatase), *pho84* (phosphate transporter), and *tgp1* (glycerophosphodiester transporter) are repressed under phosphate-replete conditions by upstream lncRNA-mediated transcriptional interference (24) and derepressed during phosphate starvation when synthesis of the interfering lncRNAs is turned off (25). *PHO* lncRNA 3'-processing and termination is a key control point in *PHO* mRNA repression; i.e., transcriptional interference can be tuned by increasing or decreasing the frequency with which Pol2 terminates lncRNA transcription prior to encounter with the mRNA promoter (24). Genetic maneuvers that enhance precocious termination of lncRNA transcription result in derepression of *PHO* mRNA expression in phosphate-replete cells, and those that reduce the probability of lncRNA termination prior to the mRNA promoter result in hyper-repression of the flanking *PHO* mRNAs relative to their basal levels (24). Because inactivating mutations of the Asp1 pyrophosphatase or deletion of the Aps1 pyrophosphatase derepress the *PHO* genes under phosphate-replete conditions and because active site mutation of the Asp1 kinase hyper-represses *pho1*, it was surmised that 1,5-IP_8_ (or 1-IP_7_) acts as an agonist of precocious *PHO* lncRNA transcription termination (22, 24).

An allelic series of Asp1 pyrophosphatase mutants—the *asp1-STF* alleles—was found to result in a severe growth defect on YES medium that results from overexpression of the *tgp1* gene and the deleterious import of glycerophosphocholine (GPC) present in the yeast extract component of the growth medium (26, 27). The *asp1-STF* strains grew on ePMGT, an enriched synthetic medium lacking GPC (27). Screening for spontaneous suppressors of the *asp1-STF* growth defect on YES identified a *tgp1*-inactivating mutation and a series of loss-of-function and hypomorphic mutations in the fission yeast 3'-processing/termination machinery, thereby fortifying the hypothesis that inositol pyrophosphate toxicosis in these strains is a consequence of precocious termination impacting Tgp1 expression (27). The synthetic lethality of *siw14*∆ *aps1*∆ could also be suppressed by *tgp1*∆ (21) and by a variety of mutations in the 3'-processing/termination machinery (12).

By contrast, the deleterious effects of combining *asp1-H397A* and *aps1*∆ could not be overcome by *tgp1*∆, which suggested that inositol pyrophosphate levels in this double-pyrophosphatase mutant exceeded a threshold beyond which overzealous 3'-processing/termination affecting genes other than *tgp1* resulted in toxicity (21). Consistent with this idea, we found that *aps1*∆ *asp1-H397A* toxicity was partially suppressed by the *ssu72-C13S* allele encoding a catalytically inactive version of the Ssu72 CTD phosphatase subunit of the Cleavage and Polyadenylation Factor (CPF) complex (22). *aps1*∆ *asp1-H397A* toxicity was suppressed fully by deletion of Spx1, a fission yeast protein composed of an inositol pyrophosphate-sensing SPX domain and a RING-finger E3 ubiquitin ligase domain (28).

The new availability of fission yeast strains in which inositol pyrophosphatases are inactivated in tandem (and that grow well by virtue of acquiring extragenic suppressors not directly involved in inositol pyrophosphate metabolism) sets the stage for the present study of inositol pyrophosphate metabolism and toxicosis, in which we demonstrate by CE-ESI-MS the synergistic impact of toxic pyrophosphatase mutations on the intracellular levels of inositol pyrophosphates and IP_6_. We find that the Asp1 and Aps1 pyrophosphatases collaborate to catabolize the inositol-1-pyrophosphates, such that their simultaneous inactivation in a *tgp1*∆ or *ssu72-C13S* background leads to huge overaccumulation of 1-IP_7_ and a partial depletion of 5-IP_7_ and IP_6_. By contrast, the Aps1 and Siw14 pyrophosphatases together catabolize the inositol-5-pyrophosphates, and their simultaneous inactivation in a suppressor background results in overaccumulation of 5-IP_7_.

To expand our knowledge of the agents and targets of inositol pyrophosphate toxicosis, we conducted a genetic screen for suppression of the severe growth defect of the *asp1-H397A aps1*∆ *tgp1*∆ strain. Analysis of eight independent *Hat* (*asp1-H397A aps1*∆ *tgp1*∆) suppressors revealed four distinct means of circumventing 1,5-IP_8_/1-IP_7_ toxicity, via (i) a missense mutation of the Swd22 subunit of CPF; (ii) missense or frameshift mutation of Spx1; (iii) novel missense mutations of the essential Kcs1 kinase that converts IP_6_ to 5-IP_7_; and (iv) inactivation of the nuclear poly(A) binding protein Nab2.

## RESULTS

### Effects of tandem Aps1 Asp1 and Siw14 Aps1 pyrophosphatase mutations on cellular inositol pyrophosphate levels

**Fig 2:**
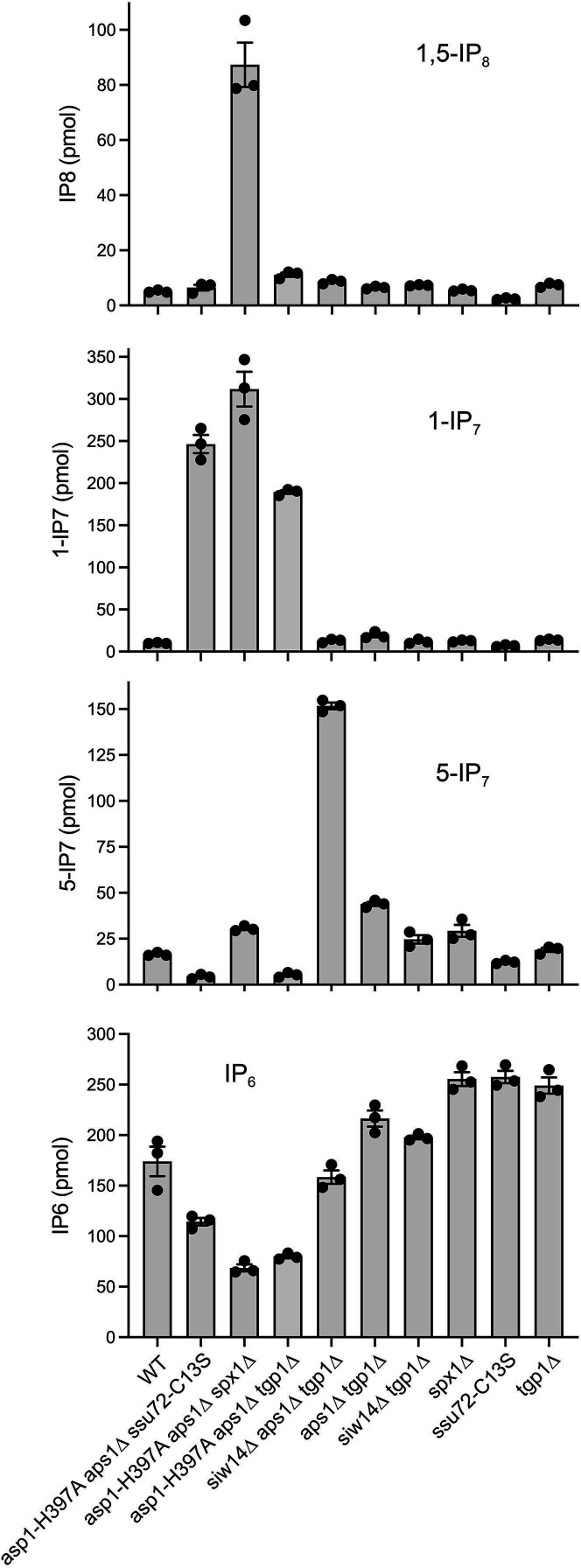
Mutational effects on inositol pyrophosphate levels as gauged by CE-ESI-MS. The amounts (pmol) of 1,5-IP_8_, 5-IP_7_, 1-IP_7_, and IP_6_ in equal volumes of extracts of the indicated fission yeast strains (from 10 *A*_600_ units of cells) are plotted. The bar heights depict the mean of three individual biological replicates (denoted by dots) ±SEM.

The advent of sensitive capillary electrophoresis electrospray ionization mass spectrometry (CE-ESI-MS) methods to profile the pool of cellular IP_6_, 5-IP_7_, 1-IP_7_, and 1,5-IP_8_ (29–31) enables us to gauge how mutations of enzymes that catabolize these molecules affect inositol pyrophosphate dynamics *in vivo*. Whole-cell perchloric acid extracts from 10 *A*_600_ units of wild-type and mutant fission yeast cells growing logarithmically in ePMGT medium at 30°C were adsorbed to titanium dioxide beads to enrich for inositol polyphosphates (32), which were eluted with 3% ammonium hydroxide. CE-ESI-MS was performed as described (29) on three biological replicates (independent cultures) of each fission yeast strain. The amounts (in pmol) of IP_6_, 5-IP_7_, 1-IP_7_, and 1,5-IP_8_ in each sample were determined and are plotted in Fig. 2 as the average of three biological replicates ±SEM. As described below, the analysis reveals that the tandem *asp1-H397A aps1*∆ and *siw14*∆ *aps1*∆ mutations elicit radically different effects on inositol pyrophosphate levels.

To characterize the impact of the tandem *asp1-H397A aps1*∆ mutations, we profiled three different strains: the “sick” *asp1-H397A aps1*∆ *tgp1*∆ triple mutant, the *asp1-H397A aps1*∆ *spx1*∆ triple mutant that grows as well as wild-type, and the partially suppressed *asp1-H397A aps1*∆ *ssu72-C13S* triple mutant. In all three strain backgrounds, we noted a huge increase in the level of 1-IP_7_ compared to the wild-type control, by 19-fold in *asp1-H397A aps1*∆ *tgp1*∆ cells (*P* value 0.0001), 31-fold in *asp1-H397A aps1*∆ *spx1*∆ cells (*P* value 0.0046), and 24-fold in *asp1-H397A aps1*∆ *ssu72-C13S* cells (*P* value 0.0021) (Fig. 2). The increase in 1-IP_7_ was accompanied by a decrease in the level of IP_6_ compared to the wild-type, by 54% in *asp1-H397A aps1*∆ *tgp1*∆ cells (*P* value 0.022), 60% in *asp1-H397A aps1*∆ *spx1*∆ cells (*P* value 0.015), and 34% in *asp1-H397A aps1*∆ *ssu72-C13S* cells (*P* value 0.047) (Fig. 2). Indeed, 1-IP_7_ was more abundant than IP_6_ in each of these three strains. By contrast, the levels of 5-IP_7_ were either lower than those of the wild-type by 68% in *asp1-H397A aps1*∆ *tgp1*∆ cells (*P* value 0.0004) and by 74% in *asp1-H397A aps1*∆ *ssu72-C13S* cells (*P* value 0.0002) or twofold higher than those of the wild-type in *asp1-H397A aps1*∆ *spx1*∆ cells (*P* value 0.0003) (Fig. 2). (Note that there are no such gross perturbations of IP_7_ levels in *tgp1*∆, *spx1*∆, or *ssu72-C13S* single mutants [Fig. 2].)

These findings underscore the following themes: (i) that the Asp1 pyrophosphatase, which specifically hydrolyzes the 1-β-phosphate, and the Aps1 pyrophosphatase, which has a modest preference for hydrolysis of the 1-β-phosphate, play essential and overlapping roles in protecting fission yeast against the accumulation of toxic levels of 1-IP_7_; (ii) that Siw14, the inositol pyrophosphatase remaining in *asp1-H397A aps1*∆ cells, is presumably responsible for generating the 1-IP_7_ via its removal of the 5-β-phosphate of 1,5-IP_8_ (Fig. 1); and (iii) the depletion of IP_6_ in lockstep with overaccumulation of 1-IP_7_ highlights the catabolism of 1-IP_7_ by Asp1 and Aps1 as a significant factor in IP_6_ homeostasis.

With respect to 1,5-IP_8_, one might have expected, according to the scheme in Fig. 1, that the loss of Asp1 and Aps1 pyrophosphatase activities would lead to an expansion of the IP_8_ pool, by diminishing the conversion of 1,5-IP_8_ back to 5-IP_7_. Yet, we see that the 1,5-IP_8_ level in *asp1-H397A aps1*∆ *tgp1*∆ cells is twofold higher than that of the wild-type (*P* value 0.0088), a modest effect compared to the increase in 1-IP_7_, and is a mere 28% higher than that of the wild-type in *asp1-H397A aps1*∆ *ssu72-C13S* cells (*P* value 0.31) (Fig. 2). We envision that the increase in 1,5-IP_8_ that does occur in the *asp1-H397A aps1*∆ *tgp1*∆ and *asp1-H397A aps1*∆ *ssu72-C13S* mutants is masked by Siw14-mediated conversion of 1,5-IP_8_ to 1-IP_7_. We see quite a different effect in the *asp1-H397A aps1*∆ *spx1*∆ strain, whereby the 1,5-IP_8_ level is 17-fold higher than that of the wild-type (*P* value 0.0094) (Fig. 2). The possible reasons for the different *asp1-H397A aps1*∆ 1,5-IP_8_ profiles in the *spx1*∆ genetic background versus the *tgp1*∆ and *ssu72-C13S* contexts will be discussed.

To gauge the effect of tandem *siw14*∆ *aps1*∆ mutations, we profiled the *siw14*∆ *aps1*∆ *tgp1*∆ strain that grows as well as wild-type at 30°C (21). We noted a ninefold increase in the level of 5-IP_7_ compared to the wild-type control (*P* value < 0.0001) (Fig. 2), to the point that 5-IP_7_ and IP_6_ were nearly equivalent in *siw14*∆ *aps1*∆ *tgp1*∆ cells. Comparison to strains *aps1*∆ *tgp1*∆ (2.7-fold increased 5-IP_7_ versus wild-type; *P* value 0.0003) and *siw14*∆ *tgp1*∆ (49% increase in 5-IP_7_ versus wild-type; *P* value 0.062) (Fig. 2) indicates that combining *aps1*∆ and *siw14*∆ exerts a synergistic effect on overaccumulation of 5-IP_7_. The *siw14*∆ *aps1*∆ *tgp1*∆ mutations had only modest effects on 1-IP_7_ (26% increase versus wild-type; *P* value 0.15) and 1,5-IP_8_ (72% increase; *P* value 0.0048), which were similar to those seen in the *aps1*∆ *tgp1*∆ and/or *siw14*∆ *tgp1*∆ strains (Fig. 2). Simultaneous ablation of Siw14 and Aps1 should, according to the scheme in Fig. 1, leave the 1-β-phosphate-specific Asp1 enzyme as the only remaining inositol pyrophosphatase, thereby interdicting the conversion of 1,5-IP_8_ to 1-IP_7_ and the conversion of 5-IP_7_ to IP_6_. We envision that the latter effect is the main driver of the overaccumulation of 5-IP_7_ in *siw14*∆ *aps1*∆ cells. We would attribute the continued presence of 1-IP_7_ in *siw14*∆ *aps1*∆ cells to the ability of the Asp1 kinase to phosphorylate IP_6_ to 1-IP_7_ (5). [We cannot exclude an alternative scenario in which another inositol pyrophosphatase, as yet unidentified, converts 1,5-IP_8_ to 1-IP_7_].

### Effect of triple Aps1 Asp1 Siw14 pyrophosphatase mutations on cellular inositol pyrophosphate levels

**Fig 3:**
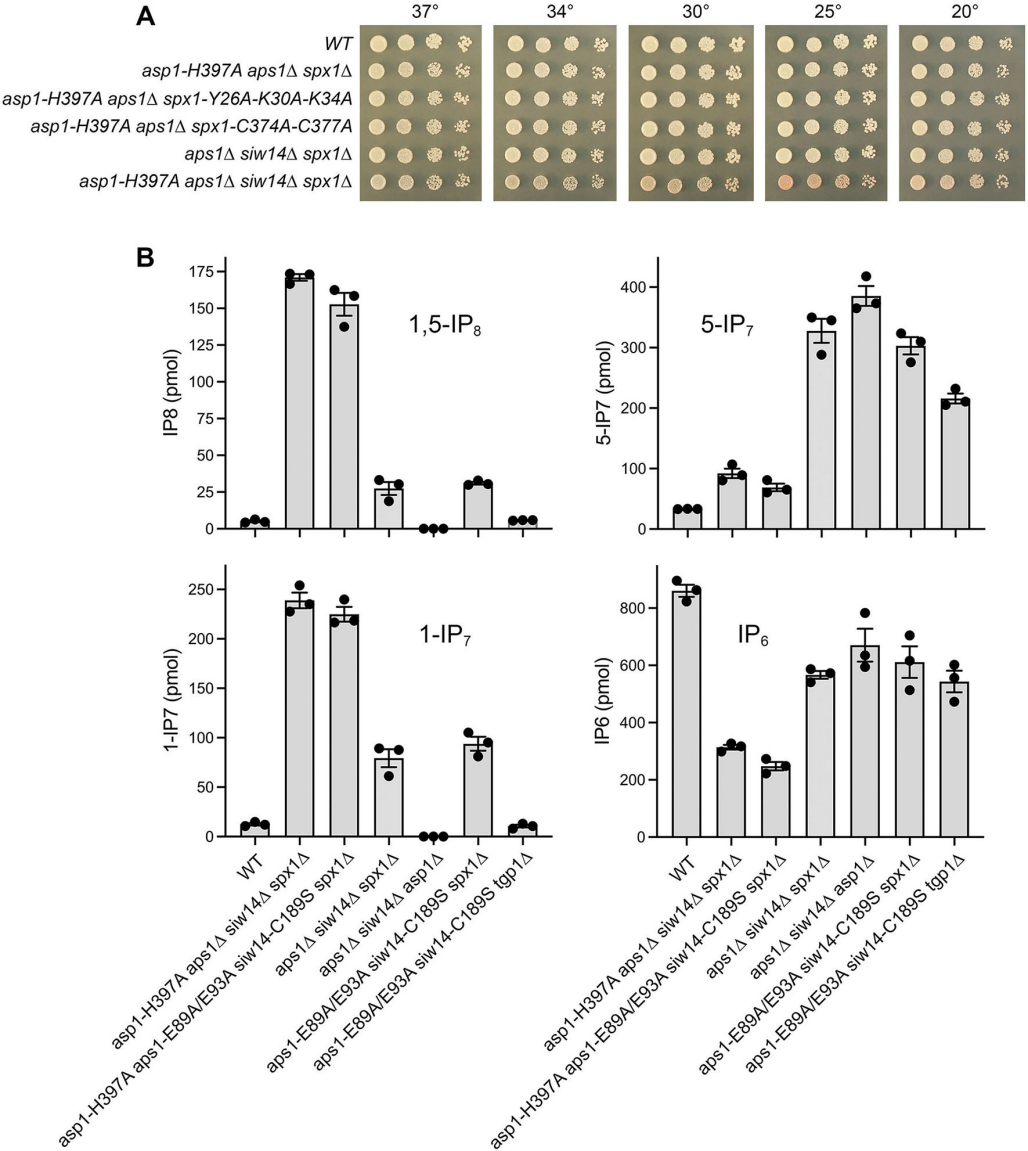
Effect of inactivating all three inositol pyrophosphatases. (**A**) Serial fivefold dilutions of fission yeast strains (as specified on the left) were spot-tested for growth on YES agar at the indicated temperatures. (**B**) The amounts (pmol) of 1,5-IP_8_, 1-IP_7_, 5-IP_7_, and IP_6_ in equal volumes of extracts of the indicated fission yeast are plotted. The bar heights depict the mean of three individual biological replicates (denoted by dots) ±SEM.

We constructed a fission yeast *asp1-H397A aps1*∆ *siw14*∆ *spx1*∆ strain in which all three known inositol pyrophosphatase enzymes were inactivated or deleted, and the ensuing toxicity on YES medium was suppressed by elimination of Spx1, i.e., the *asp1-H397A aps1*∆ *siw14*∆ *spx1*∆ mutant grew well on YES agar at all temperatures tested (Fig. 3A).

The 1,5-IP_8_, 1-IP_7_, 5-IP_7_, and IP_6_ profiles of the *asp1-H397A aps1*∆ *siw14*∆ *spx1*∆ mutant were analyzed in parallel with a wild-type control (Fig. 3B). The salient findings were that simultaneous ablation of the three pyrophosphatases elicited prodigious increases in 1,5-IP_8_ (by 33-fold versus wild-type; *P* value < 0.0001) and 1-IP_7_ (by 19-fold; *P* value 0.0009), while reducing IP_6_ by threefold (*P* value 0.0004) (Fig. 3B). By contrast, 5-IP_7_ was increased modestly (by threefold; *P* value 0.017) (Fig. 3B). The magnitudes of the changes in the *asp1-H397A aps1*∆ *siw14*∆ *spx1*∆ mutant were very similar to those observed for the *asp1-H397A aps1*∆ *spx1*∆ strain (Fig. 2).

To affirm that the inositol pyrophosphate changes in the *asp1-H397A aps1*∆ *siw14*∆ *spx1*∆ mutant were a consequence of the loss of Aps1 and Siw14 catalytic activity rather than the absence of the Aps1 and Siw14 proteins, we constructed an *asp1-H397A aps1-E89A/E93A siw14-C189S spx1*∆ strain that expresses the catalytically defective Aps1-E89A/E93A and Siw14-C189S proteins (12, 21) from the native *aps1* and *siw14* chromosomal loci. The inositol pyrophosphate profile of the *asp1-H397A aps1-E89A/E93A siw14-C189S spx1*∆ mutant echoed that of the *asp1-H397A aps1*∆ *siw14*∆ *spx1*∆ strain that lacks the Aps1 and Siw14 proteins (Fig. 3B).

**Fig 4:**
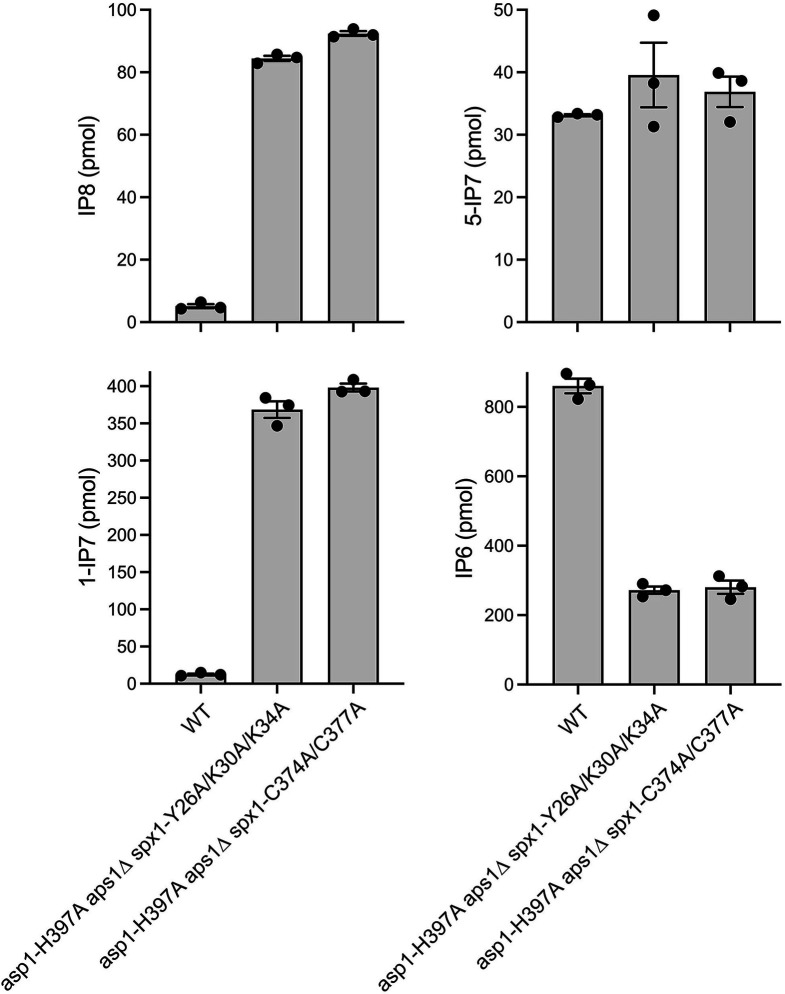
SPX domain and RING domain mutations echo *spx1*∆ with respect to inositol pyrophosphate changes. The amounts (pmol) of 1,5-IP_8_, 5-IP_7_, 1-IP_7_, and IP_6_ in equal volumes of extracts of fission yeast strains—wild-type (WT), *asp1-H397A aps1∆ spx1-Y26A/K30A/K34A*, and *asp1-H397A aps1∆ spx1-C374A/C377A*—are plotted. The bar heights depict the mean of three individual biological replicates (denoted by dots) ± SEM. Note that the inositol pyrophosphate and IP_6_ data reported in Fig. 4 and 3B are from the same experiment; consequently, the wild-type control values are the same in both figures.

In addition, we queried the potential effects of mutations in the SPX and RING domains of Spx1 on the changes in inositol pyrophosphates, especially the increase in 1,5-IP_8_, elicited by tandem inactivation of the Asp1 and Aps1 pyrophosphatases. We verified that the *asp1-H397A aps1*∆ growth defect is suppressed by a *Y26A-K30A-K34A* mutation in the inositol pyrophosphate binding site of the SPX domain of Spx1 or a *C374A-C377A* mutation in the zinc-binding site of the ubiquitin ligase domain of Spx1, thus phenocopying the suppression by *spx1*∆ (Fig. 3A). The *asp1-H397A aps1*∆ alleles in the context of the Spx1 SPX and RING mutations resulted in 16-fold (*P* value < 0.0001) and 18-fold (*P* value < 0.0001) elevations in 1,5-IP_8_ versus wild-type, respectively, and 29-fold (*P* value 0.0009) and 32-fold (*P* value < 0.0001) elevations in 1-IP_7_, respectively, while reducing IP_6_ by threefold (*P* values 0.0002 and <0.0001, respectively), and having a minimal effect on 5-IP_7_ (*P* values 0.34 and 0.27, respectively) (Fig. 4). The directions and magnitudes of these changes were very similar to those observed for the *asp1-H397A aps1*∆ *spx1*∆ strain (Fig. 2).

### Transcriptome profile of *asp1-H397A aps1*∆ *siw14*∆ *spx1*∆ cells

Transcriptional profiling of *asp1-H397A* and *aps1*∆ cells delineated an inositol pyrophosphate-responsive regulon composed of genes overexpressed when 1,5-IP_8_/1-IP_7_ levels are increased (22). The mRNAs most affected by *asp1-H397A* were four phosphate-regulated genes driven by transcription factor Pho7 (23, 25, 31), these being *tgp1* (up 21-fold), *ecl3* (up sevenfold), *pho1* (up sevenfold), and *pho84* (up fourfold) (22). These same four transcripts were “top hits” for upregulation in *aps1*∆ cells, as was the phosphate/Pho7-regulated *efn1* gene encoding an extracellular 5'-nucleotidase (22, 25, 33, 34), which was upregulated sixfold in the *aps1*∆ strain. Moreover, *pho1*, *pho84*, *efn1*, and *ecl3* were all downregulated (by 20-fold, 14-fold, 7-fold, and 5-fold, respectively) in *asp1-D333A* cells that lack 1,5-IP_8_/1-IP_7_ (22). By contrast, *siw14*∆ had virtually no effect on the fission yeast transcriptome (12).

Because deleting or inactivating Spx1 completely suppresses the growth defect of cells lacking two or all three inositol pyrophosphatase activities, it was of interest to gauge whether *spx1*∆ also suppressed the transcriptional changes associated with loss of Asp1 and Aps1 pyrophosphatase activities. To that end, we performed RNA-seq on poly(A)^+^ RNA isolated from *asp1-H397A aps1*∆ *siw14*∆ *spx1*∆ cells and from wild-type controls. cDNAs obtained from three biological replicates were sequenced for each strain. We thereby detected 13 protein-coding transcripts that were upregulated by 2- to 7-fold in *asp1-H397A aps1*∆ *siw14*∆ *spx1*∆ cells and 71 mRNAs that were downregulated by twofold to 19-fold (Table S1). The salient point is that none of the phosphate-regulated genes that were upregulated in *asp1-H397A* and *aps1*∆ cells were dysregulated in *asp1-H397A aps1*∆ *siw14*∆ *spx1*∆ cells. Thus, deletion of Spx1 blunts the signature effects of increased 1,5-IP_8_/1-IP_7_ on fission yeast gene expression.

### Effects of inactivating Aps1 and Siw14 in cells lacking Spx1 versus Tgp1

We proceeded to examine the impact of the *aps1*∆ *siw14*∆ double deletion in the context of the *spx1*∆ allele that allows growth on YES medium at all temperatures (Fig. 3A). This analysis was performed in parallel with an *aps1-E89A/E93A siw14-C189S spx1*∆ mutant that expresses phosphatase-defective Aps1 and Siw14 proteins and with an *aps1-E89A/E93A siw14-C189S tgp1*∆ strain. (Note that *tgp1*∆ is a less effective suppressor of *aps1*∆ *siw14*∆ compared to *spx1*∆ insofar as the *aps1*∆ *siw14*∆ *tgp1*∆ strain does not grow on YES medium at 37˚C [21].) The *aps1*∆ *siw14*∆ *spx1*∆ cells evinced a fivefold increase in 1,5-IP_8_ versus wild-type (*P* value 0.035) and a sixfold increase in 1-IP_7_ (*P* value 0.017) (Fig. 3B). The same increases in 1,5-IP_8_ and 1-IP_7_ were observed in the *aps1-E89A/E93A siw14-C189S spx1*∆ strain, but not in the *aps1-E89A/E93A siw14-C189S tgp1*∆ strain (Fig. 3B). By contrast, 5-IP_7_ levels were increased in all three strains that lacked Aps1 and Siw14 pyrophosphatase activities: by 10-fold in *aps1*∆ *siw14*∆ *spx1*∆ cells (*P* value 0.0045), ninefold in *aps1-E89A/E93A siw14-C189S spx1*∆ cells (*P* value 0.0028), and sevenfold in *aps1-E89A/E93A siw14-C189S tgp1*∆ cells (*P* value 0.002) (Fig. 3B).

The findings regarding *aps1-E89A/E93A siw14-C189S tgp1*∆ cells, taken together with those above that *siw14*∆ *aps1*∆ *tgp1*∆ cells have ninefold higher 5-IP_7_ and little or no change in 1,5-IP_8_ and 1-IP_7_ (Fig. 2), raise the question of whether the severe growth defect of *aps1*∆ *siw14*∆ cells on YES medium is caused solely by a putative toxic effect of too much 5-IP_7_. Two observations militate against this scenario. First, we have shown that the synthetic lethality of *aps1*∆ *siw14*∆ depends on the synthesis of 1,5-IP_8_ by the Asp1 kinase, i.e., that an *aps1*∆ *siw14*∆ *asp1-D333A* strain that expresses a kinase-dead Asp1 protein grows as well as a wild-type strain on YES medium at all temperatures (12). Second, we find here that *aps1*∆ *siw14*∆ *asp1*∆ cells have no detectable 1,5-IP_8_ or 1-IP_7_ while manifesting a 12-fold increase in 5-IP_7_ (Fig. 3B). We conclude that elevated 5-IP_7_ does not suffice for the toxicity of *aps1*∆ *siw14*∆, which depends on the presence of 1,5-IP_8_/1-IP_7_.

### Spontaneous suppressors of *asp1-H397A aps1*∆ *tgp1*∆ toxicosis

**Fig 5:**
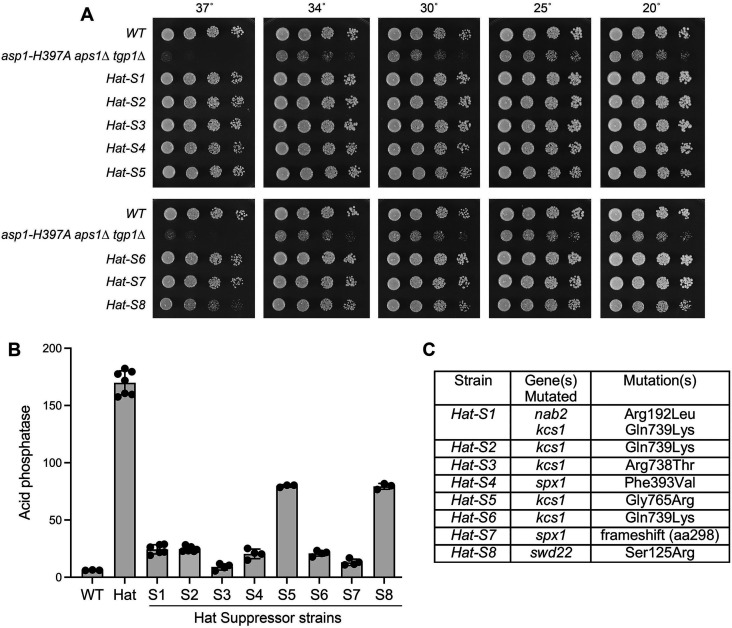
Isolation of spontaneous suppressors of *asp1-H397A aps1*∆ *tgp1*∆ toxicosis. (**A**) Serial fivefold dilutions of fission yeast strains—wild-type (WT), *asp1-H397A aps1*∆ *tgp1*∆ (Hat), and eight independent Hat-suppressor strains (*Hat-S* series)—were spot-tested for growth on YES agar at the indicated temperatures. (**B**) The indicated strains were grown to *A*_600_ of 0.5 to 0.8 in liquid culture in ePMGT medium at 30°C. Cells were then harvested, washed with water, and assayed for Pho1 acid phosphatase activity. (**C**) Whole-genome sequencing of the *Hat-S* strains revealed the indicated mutations.

We reported recently that *asp1-H397A aps1*∆ *tgp1*∆ cells are sick at 20–34°C and fail to form colonies at 37°C (21) (Fig. 5A). This phenotype afforded us an opportunity to identify determinants of 1,5-IP_8_/1-IP_7_ toxicosis that act independently of the Tgp1 overexpression that underlies the growth defect of the *asp1-STF* strains (27). We screened for candidate *Hat* (*asp1-H397A aps1*∆ *tgp1*∆) suppressor mutants by (i) inoculating single colonies of fresh *asp1-H397A aps1*∆ *tgp1*∆ haploid cells in 4 mL of YES liquid medium and incubating for 24 hours at 30°C; (ii) diluting 10 µL aliquots of each culture into 5 mL of fresh YES medium and incubating for 24 hours at 30°C; (iii) repeating the dilution and 24 hour incubation steps; and (iv) streaking aliquots of each culture on YES agar plates and selecting rare single colonies that grew to large size against a background of tiny colonies. One large colony isolate derived from each of the original starting *asp1-H397A aps1*∆ *tgp1*∆ cultures was picked for further analysis. Eight independent *Hat-S* (Hat-Suppressor) strains were grown and re-streaked for single colonies at 30°C, which were homogeneously larger than the parental *asp1-H397A aps1*∆ *tgp1*∆ (*Hat*) strain. Spot testing of serial dilutions of cells for growth on YES agar showed that all eight *Hat-S* isolates grew as well as wild-type at 25°C, 30°C, and 34°C, as gauged by colony size (Fig. 5A). In 7/8 *Hat-S* strains, the inability of *asp1-H397A aps1*∆ *tgp1*∆ cells to form colonies at 37°C was fully reversed, as was the slow-growth defect of *asp1-H397A aps1*∆ *tgp1*∆ cells at 20°C. By contrast, the *Hat-S8* suppressor formed tiny colonies at 37°C and partially restored growth at 20°C (Fig. 5A).

Assay of the fission yeast strains for cell surface Pho1 acid phosphatase activity during exponential growth at 30°C in phosphate-replete ePMGT medium showed that Pho1 expression was 28-fold higher in *asp1-H397A aps1*∆ *tgp1*∆ (*Hat*) cells vis-à-vis wild-type cells (Fig. 5B). Pho1 expression was restored to near-wild-type basal levels in the *Hat-S3* and *Hat-S7* strains and was reduced to varying degrees in the other *Hat-S* strains compared to the fully derepressed *asp1-H397A aps1*∆ *tgp1*∆ parent (Fig. 5B). Based on the Pho1 assays and the growth profiles, we regard *Hat-S8* as the weakest of the suppressor isolates.

Paired-end Illumina sequencing of unamplified genomic DNA from the *Hat-S* strains was performed. All the *Hat-S* isolates retained their original *asp1-H397A* alleles. The genomic mutations identified for each isolate are compiled in Fig. 5C. The mutated proteins fall into several functional categories: (i) Swd22, a subunit of CPF; (ii) Spx1, an inositol pyrophosphate sensor that transduces the signal for precocious 3'-processing/termination; and (iii) Kcs1, an essential enzyme of 5-IP_7_ biogenesis.

#### CPF subunit Swd22

Strain *Hat-S8* contains a single Ser125Arg missense mutation in the inessential *swd22* gene, which encodes the 341-aa Swd22 subunit of CPF. Swd22 consists of seven tandem WD β-propeller domains. Swd22, Dis2 (a phosphoprotein phosphatase), and Ppn1 (a regulator of Dis2 phosphatase) comprise a three-subunit DPS module within the 13-subunit CPF complex (35). An AlphaFold 3 model (36) of a heterotrimeric DPS complex (not shown) accurately places the two metal ions in the Dis2 active site and appears to correctly situate the mapped interfaces between Ppn1 and Dis2 and Ppn1 and Swd22 (37). Swd22 Ser125 is located within the β2 strand of the third β-propeller. As modeled, the Ser125-OH makes bifurcated hydrogen bonds to His107-Nε and Trp135-Nε. Replacing Ser125 with a bulky arginine side chain would likely disrupt the structure of this WD repeat. Previous studies established a crucial connection between Swd22 and 1,5-IP_8_/1-IP_7_ status, insofar as (i) *asp1*∆ and *asp1-D333A* are synthetically lethal with *swd22*∆, signifying that 1,5-IP_8_/1-IP_7_ synthesized by Asp1 kinase and Swd22 play important but genetically redundant roles in promoting essential 3'-processing/transcription termination events; (ii) de-repression of *pho1* by *asp1-H397A* and *aps1*∆ is erased by *swd22*∆; and (iii) the lethality of *asp1-H397A aps1*∆ is suppressed by *swd22*∆, even at 37°C (22). We surmise that the *swd22-S125R* allele identified in the *Hat* suppressor screen is a loss-of-function variant that affirms the role of CPF as the mediator of inositol pyrophosphate toxicosis.

#### Spx1

**Fig 6:**
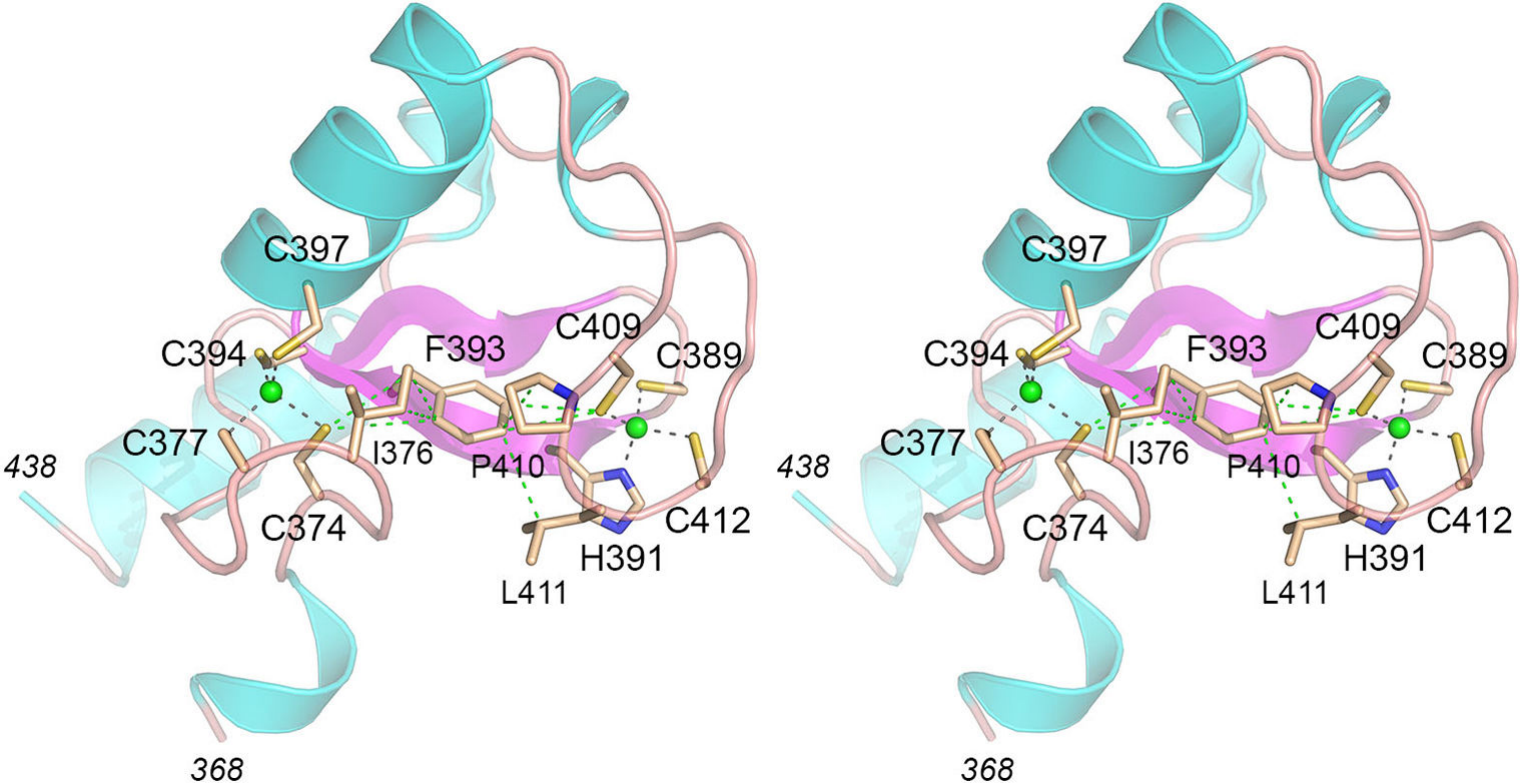
Predicted role of Spx1 Phe393 in the Zn-binding RING domain. Stereo view of an AlphaFold 3 model of the Spx1 RING domain in complex with two zinc atoms (green spheres). The atomic contacts between the Cys and His side chains that tetrahedrally coordinate the Zn atoms are denoted by black dashed lines. Phe393, the residue mutated to Val in the *Hat-S4* strain, is situated midway between the two Zn complexes. van der Waals contacts between Phe293 and nearby amino acids are denoted by green dashed lines.

The recovery of two new alleles of *spx1* in the *Hat-S* screen fortifies the already solid case for Spx1 (a 470-aa protein) as the key inositol pyrophosphate-binding sensor that connects elevated IP_8_/1-IP_7_ status to precocious 3'-processing/termination. The mutation in *Hat-S7* is a 20-nucleotide deletion starting in the Leu298 codon that results in a translation frameshift that appends a foreign octapeptide (SCFFSANE) following Glu297 before encountering a new stop codon. The effect of this mutation is to eliminate the C-terminal Zn-binding RING domain and to truncate the N-terminal SPX domain. The mutation in *Hat-S4* is a Phe393Val missense change. An AlphaFold 3 model of Spx1 places two Zn^2+^ ions in the RING domain, in tetrahedral coordination complexes with Cys374, Cys377, Cys394, Cys397 and Cys389, His391, Cys409, and Cys412 (Fig. 6). Phe393 is predicted to sit midway between the two Zn^2+^ coordination complexes and to make multiple van der Waals contacts to nearby aliphatic side chains Ile376, Pro410, and Leu411 as well as to Cys374 and Cys409 (Fig. 6). We speculate that the replacement of Phe293 by Val will sever the contacts to Cys409, Pro410, and Leu411 and thereby affect the stability and/or function of the RING domain and its imputed ubiquitin ligase activity.

#### IP_6_ kinase Kcs1

Here, we recovered three new missense alleles of *kcs1—Q739K* (in two independent isolates *Hat-S2* and *Hat-S6*), *R738T* (in *Hat-S3*), and *G765R* (in *Hat-S5*)—that completely suppressed the growth defect and reversed the *pho1* derepression of the *asp1-H397A aps1*∆ *tgp1*∆ mutant (Fig. 5). Previously, we garnered three other *kcs1* missense alleles*—R332T*, *L338R*, and *E834K*—that completely reversed the *asp1-STF* growth defect and one allele *S761F* that partially suppressed (27). Collectively, our genetic findings, supported by inositol pyrophosphate profiling of *kcs1-R332T* cells (21), suggest that a reduction in the kinase activity of the hypomorphic mutant Kcs1 enzymes limits the pool of 5-IP_7_ available for conversion to 1,5-IP_8_ by the pyrophosphatase-defective Asp1-STF or Asp1-H397A kinase.

**Fig 7:**
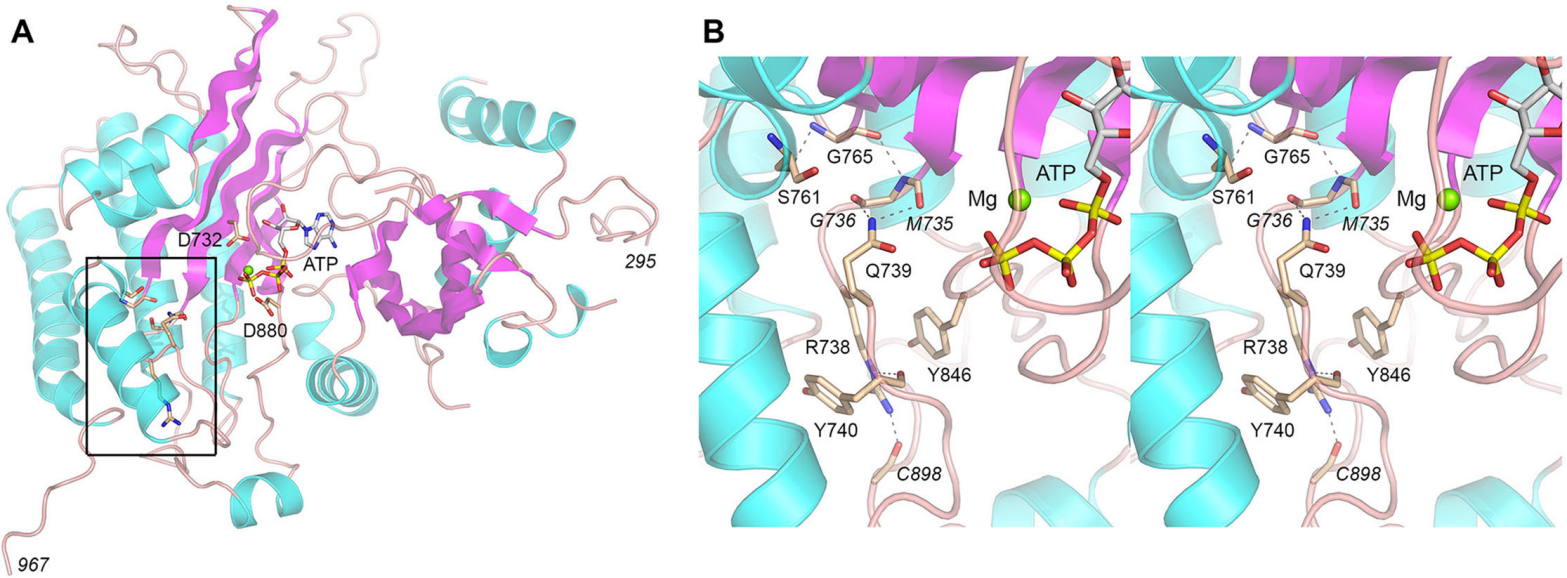
Kcs1 mutations that suppress the inositol pyrophosphate toxicity cluster within the IP_6_ kinase domain. (**A**) AlphaFold 3 model of the Kcs1 kinase domain in complex with ATP (stick model) and Mg^2+^ (green sphere). The amino acids that are mutated in the *Hat-S* suppressor strains colocalize within the region flanking the ATP site, demarcated by the box. (**B**) Stereo view of the predicted atomic interactions of the amino acids that are mutated in *kcs1* alleles that suppress inositol pyrophosphate toxicosis.

To gain insights into how the suppressor mutations might impact IP_6_ kinase activity, we generated an AlphaFold 3 model of Kcs1 in complex with ATP and Mg^2+^. Much of the 967-aa Kcs1 polypeptide consists of intrinsically disordered segments (aa 1–294, 376–410, 433–469, and 509–706). The remainder of the Kcs1 polypeptide adopts an inositol polyphosphate kinase fold that accommodates ATP•Mg^2+^ in the phosphate donor site formed by two β-sheets, wherein the divalent cation is coordinated by Asp880 and the ATP phosphates, and Asp732 engages the ATP ribose 3′-OH (Fig. 7A). The Kcs1 kinase domain model is homologous to the crystal structure of *Entamoeba histolytica* IP6K (38). The instructive findings here are that each of the three suppressor mutants identified presently (R738T, Q739K, and G765R), as well as the S761F suppressor mutant reported earlier, map to amino acids that are closely clustered within the IP_6_ kinase domain in a region adjacent to the phosphate donor site (circumscribed by the box in Fig. 7A). A close-up stereo view of the region (Fig. 7B) highlights a network of atomic contacts by and among these amino acids, as follows. Gln739 makes bifurcated hydrogen bonds to the mainchain carbonyls of Met735 and Gly736. Gly765 donates a hydrogen bond from its amide to the Ser761 carbonyl and accepts a hydrogen bond to its carbonyl from the Met735 amide (Fig. 7B). This network would be perturbed by installation of larger side chains Lys, Arg, and Phe in lieu of Gln739, Gly765, and Ser761, respectively. Arg738 is sandwiched in π–cation–π stack between Tyr740 and Tyr846 and makes a hydrogen bond with the Cys898 carbonyl (Fig. 7B). These interactions would not be sustained when Thr replaces Arg738. Reference to the crystal structure of *Entamoeba* IP6K in complex with IP_6_ and ATP (38) indicates that this cluster of amino acids is located underneath the binding pocket for IP_6_. Thus, we speculate that the hypomorphic Kcs1 mutations that suppress inositol pyrophosphate toxicosis might negatively impact Kcs1 interaction with its IP_6_ phosphoacceptor substrate.

### *nab2*∆ suppresses *asp1-H397A aps1*∆ *tgp1*∆ toxicosis

**Fig 8:**
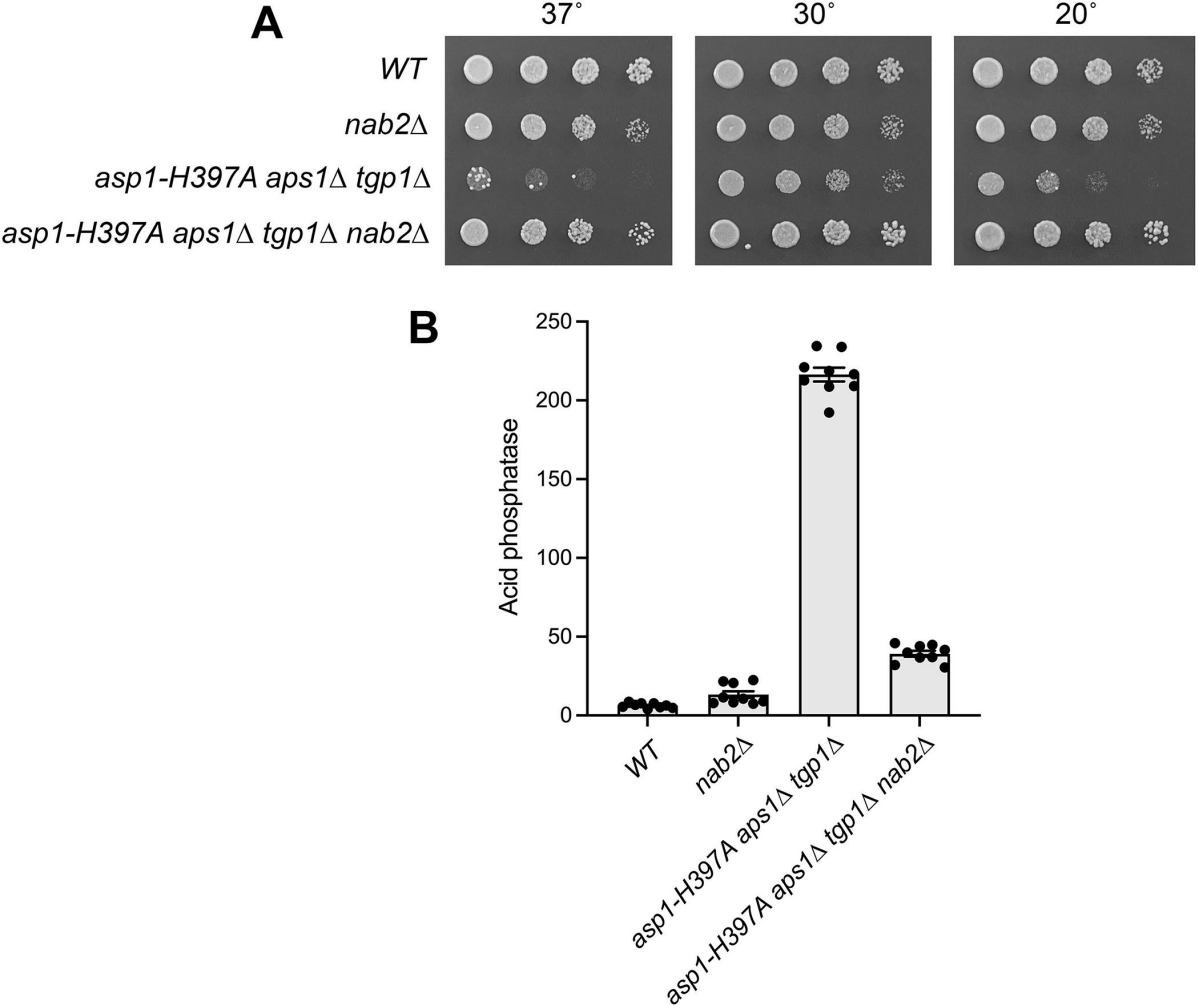
*nab2*∆ suppresses *asp1-H397A aps1*∆ *tgp1*∆ toxicosis. (**A**) Serial fivefold dilutions of the indicated fission yeast strains were spot-tested for growth on YES agar at the indicated temperatures. (**B**) The indicated yeast strains were grown to *A*_600_ of 0.5 to 0.8 in liquid culture in YES medium at 30°C. Cells were then harvested, washed with water, and assayed for Pho1 acid phosphatase activity.

The genome of the *Hat-S1* strain acquired two mutations: *kcs1-Q739K* (that suffices for suppression in *Hat-S2* and *Hat-S6*) and a missense mutation *nab2-R192L* in the gene encoding Nab2, a 307-aa nuclear poly(A) binding protein. Nab2, which is inessential in fission yeast, consists of an N-terminal PWI-fold domain and three CCCH-type Zn finger modules (39). An AlphaFold 3 model of Nab2 in complex with three Zn^2+^ ions reveals an N-terminal domain comprising a five-helix bundle (aa 1–92) and three tandem Zn fingers (aa 182–270); the rest of the protein is predicted to be disordered. The three Zn fingers of fungal Nab2 are responsible and sufficient for poly(A) binding (40). Arg192, the residue mutated in *Hat-S1*, is located in the first Zn finger module, between Zn-binding residues Cys190 and Cys195. We considered two scenarios regarding the *nab2-R192L* mutation, whereby (i) it is incidental and unrelated to *Hat* suppression; or (ii) it contributes to *Hat* suppression, potentially arising first to confer a modest advantage that was consolidated by the *kcs1-Q739K* mutation. As a direct probe of the potential role of Nab2 in inositol pyrophosphate toxicosis, we constructed an *nab2*∆ strain (in which the *nab2*^+^ gene was replaced with a *kanMX* cassette) and crossed it to the *asp1-H397A aps1*∆ *tgp1*∆ strain. The resulting *asp1-H397A aps1*∆ *tgp1*∆ *nab2*∆ strain grew as well as a wild-type strain at 20°C, 30°C, and 37°C (Fig. 8A). Moreover, the introduction of *nab2*∆ into the *asp1-H397A aps1*∆ *tgp1*∆ mutant substantially reduced the derepression of Pho1 acid phosphatase activity elicited by inositol pyrophosphate excess (Fig. 8B). These results implicate Nab2 as a newly appreciated agent of inositol pyrophosphate toxicity.

## DISCUSSION

**Fig 9:**
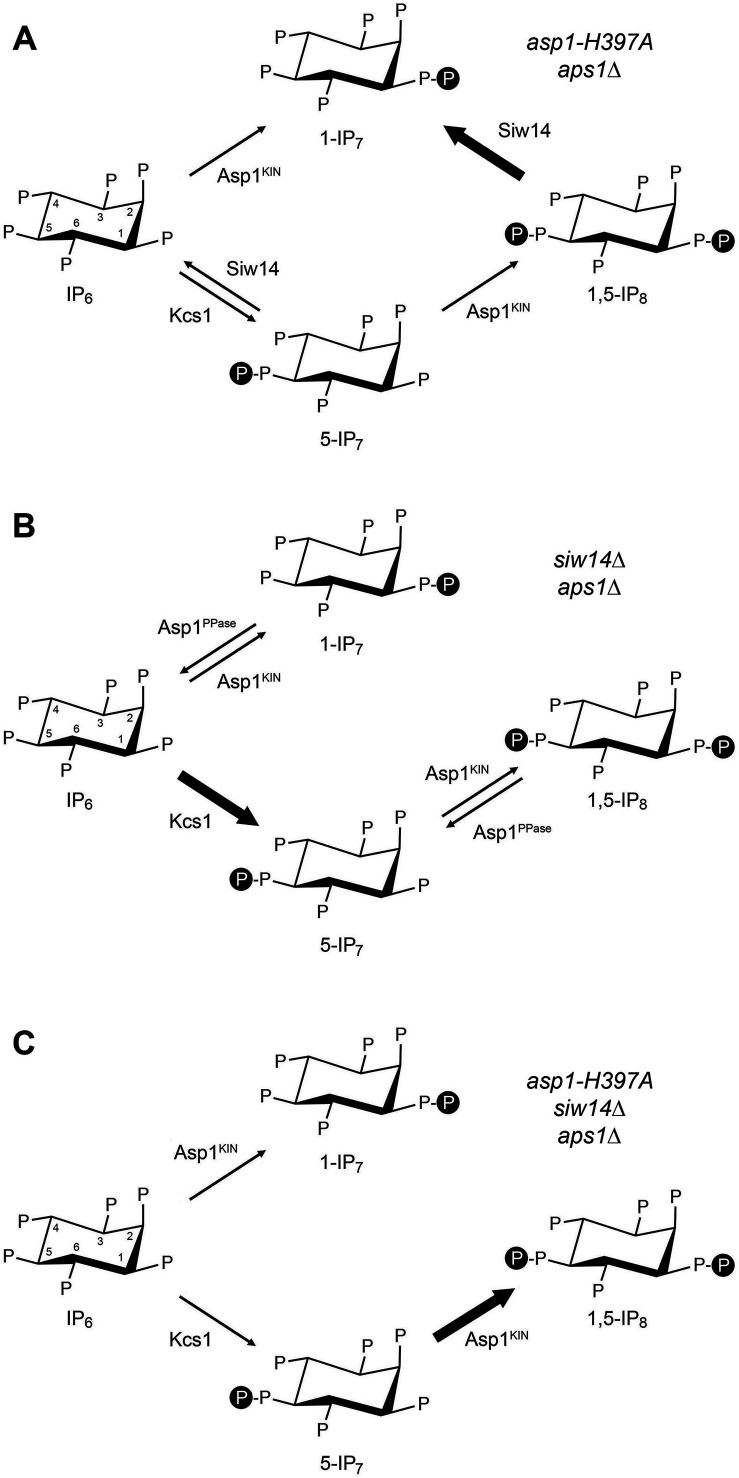
Summary of changes in inositol pyrophosphate metabolism in cells lacking two or more inositol pyrophosphatases. (**A**) Cells lacking Asp1 and Aps1 pyrophosphatases. (**B**) Cells lacking Siw14 and Aps1 pyrophosphatases. (**C**) Cells lacking Asp1, Siw14, and Aps1 pyrophosphatases.

The studies herein extend our knowledge of inositol pyrophosphate metabolism and the basis for inositol pyrophosphate toxicosis in fission yeast. By applying CE-ESI-MS to profile the inositol pyrophosphate and IP_6_ content of fission yeast mutants, we shed light on the functional redundancies of the Asp1, Siw14, and Aps1 pyrophosphatases in their catabolism of inositol pyrophosphates. Asp1, which exclusively cleaves the 1-β-phosphate, and Aps1, which has a preference for cleavage of the 1-β-phosphate, play essential overlapping roles in guarding against the accumulation of toxic levels of 1-IP_7_. In *asp1-H397A aps1*∆ cells in which toxicity is suppressed by *tgp1*∆ or *ssu72-C13S*, we observed an ~20-fold increase in 1-IP_7_ and a partial depletion of 5-IP_7_ and IP_6_. We surmise that Siw14, the inositol pyrophosphatase remaining in *asp1-H397A aps1*∆ cells, generates the 1-IP_7_ increase via its removal of the 5-β-phosphate of 1,5-IP_8_ (Fig. 9A). Accordingly, we see only a modest twofold increase in 1,5-IP_8_ in *asp1-H397A aps1*∆ *tgp1*∆ cells. These findings suggest that a supra-threshold accumulation of 1-IP_7_ is toxic to fission yeast. Note that the depletion of IP_6_ coupled with overaccumulation of 1-IP_7_ highlights the catabolism of 1-IP_7_ by Asp1 and Aps1 as a significant factor in IP_6_ homeostasis (Fig. 9A).

The synthetic lethality of *aps1*∆ *siw14*∆ is suppressed by *tgp1*∆. In this context, simultaneous loss of the Aps1 and Siw14 pyrophosphatases results in a synergistic (ninefold) increase in 5-IP_7_ vis-à-vis the modest effects on 5-IP_7_ seen in *aps1*∆ *tgp1*∆ and *siw14*∆ *tgp1*∆ strains. With the 1-β-phosphate-specific Asp1 enzyme being the sole inositol pyrophosphatase in *aps1*∆ *siw14*∆ *tgp1*∆ cells, there is no catabolism of inositol-5-pyrophosphates, i.e., there is no conversion of 1,5-IP_8_ to 1-IP_7_ or of 5-IP_7_ to IP_6_ (Fig. 9B). The fact that 1-IP_7_ is present in *aps1*∆ *siw14*∆ *tgp1*∆ cells attests to the capacity of Asp1 kinase to directly phosphorylate IP_6_ to 1-IP_7_ as an alternative route of 1-IP_7_ biogenesis (Fig. 9B), in lieu of the prevailing view that 1-IP_7_ is derived by catabolism of 1,5-IP_8_ (41). Whereas the manifold increase in 5-IP_7_ distinguishes *aps1*∆ *siw14*∆ from *asp1-H397A aps1*∆, it does not suffice for the growth defect of *aps1*∆ *siw14*∆, which requires the presence of inositol-1-pyrophosphates synthesized by the Asp1 kinase. Our results suggest that a suprathreshold level of 5-IP_7_ combined with slightly elevated (<2 fold) levels of inositol-1-pyrophosphates drives an alternative path to toxicity distinct from that elicited by the massive overaccumulation of inositol-1-pyrophosphates in *asp1-H397A aps1*∆ cells. Bear in mind that in both cases, the growth defect arises from overzealous 3'-processing/transcription termination and requires transduction of an inositol pyrophosphate signal via Spx1.

A noteworthy finding here is that the levels of 1,5-IP_8_ are significantly higher when the *asp1-H397A aps1*∆ and *aps1*∆ *siw14*∆ double mutations are profiled in an *spx1*∆ genetic background versus a *tgp1*∆ background, keeping in mind that neither *tgp1*∆ nor *spx1*∆ *per se* have an effect on inositol-1-pyrophosphate levels. Indeed, the magnitudes of the increases in 1,5-IP_8_ and 1-IP_7_ in *asp1-H397A aps1*∆ *spx1*∆ cells phenocopy those in *asp1-H397A aps1*∆ *siw14*∆ *spx1*∆ cells that lack all inositol pyrophosphatases (Fig. 9C). It is conceivable that *spx1*^+^ deletion somehow negatively impacts the activity of Siw14, thereby raising the levels of 1,5-IP_8_ (and 1-IP_7_) vis-à-vis the *tgp1*∆ contexts. One can speculate that Siw14 pyrophosphatase activity, which is inhibited by inorganic phosphate *in vitro* (12), is diminished *in vivo* in *asp1-H397A aps1*∆ *spx1*∆ cells by the increase in free intracellular phosphate that occurs when *spx1*^+^ (also known as *pqr1*^+^) is deleted (42). Similar considerations may come into play in *aps1*∆ *siw14*∆ *spx1*∆ cells, which have higher levels of 1,5-IP_8_ and 1-IP_7_ than do *aps1*∆ *siw14*∆ *tgp1*∆ cells. The residual Asp1 pyrophosphate enzyme is inhibited by inorganic phosphate, which promotes net synthesis of inositol-1-pyrophosphates by the Asp1 kinase (5).

The present conclusions that different families of inositol pyrophosphatases serve functionally important overlapping roles in physiology are likely to apply to other eukaryal taxa. For example, plants encode multiple paralogs of each of the three classes of inositol pyrophosphatase enzymes found in fission yeast: histidine acid phosphatase, Nudix hydrolase, and cysteinyl phosphatase. A recent study interrogating their potential functional redundancies in plants showed that simultaneous knockout of three Nudix-type inositol pyrophosphatases in *Arabidopsis thaliana* did not elicit an overt growth phenotype but did result in ~2-fold increases in IP_6_ and 1-IP_7_ content (43). By contrast, single knockouts of a Nudix-type inositol pyrophosphatase (MpNUDT1, which strongly prefers 1-IP_7_ as the substrate *in vitro*) or a cysteinyl phosphatase-type inositol pyrophosphatase (MpDSP1) in *Marchantia polymorpha* led to growth and development abnormalities, as did a kinase-active/pyrophosphatase-dead MpVIP1 mutant (analogous to Asp1-H397A in fission yeast) (43, 44). The MpNUDT1 mutant plant displayed an 2-fold to fivefold increase in 1-IP_7_ content (43, 44). Combining the MpDSP1 knockout with pyrophosphatase-dead MpVIP1 exacerbated some of the development phenotypes. It was not possible to recover a *Marchantia* MpNUDT1 pyrophosphatase-dead MpVIP1 double-mutant or a MpNUDT1 MpDSP1 double-mutant (43), thus echoing the synthetic lethality of *asp1-H397A aps1*∆ and *aps1*∆ *siw14*∆ in fission yeast.

Here, we conducted a new genetic screen for suppressors of the severe *asp1-H397A aps1*∆ *tgp1*∆ growth defect, which aimed to identify factors that mediate inositol pyrophosphate toxicosis independent of the *tgp1*^+^ overexpression that causes toxicity by virtue of the increased import of GPC (27). Our recovery of a novel missense mutation in the Swd22 subunit of CPF, as well as two inactivating mutations in Spx1, adds to the copious evidence that *asp1-H397A aps1*∆ toxicity is a consequence of an overdrive of inositol pyrophosphate agonism of 3'-processing/termination, mediated via the inositol pyrophosphate sensor and RING domains of Spx1 (22, 28). The *Hat-S* screen yielded three new missense mutations of Kcs1, the essential kinase that converts IP_6_ to 5-IP_7_, that restore “normal” growth on YES agar. The Kcs1 amino acids mutated in the *Hat-S* strains cluster within the kinase domain in a region close to the predicted IP_6_ substrate binding site, leading us to infer that a decrement in IP_6_ kinase activity limits the pool of 5-IP_7_ available for conversion to downstream metabolites 1,5-IP_8_ and 1-IP_7_. Testing this prediction *in vitro* has been stymied by our inability to produce recombinant Kcs1 in bacteria, likely owing to the predicted disordered state of most of the Kcs1 protein.

The occurrence in the *Hat-S1* strain of an *nab2* missense mutation along with *kcs1* missense mutation prompted us to query if Nab2 might impact inositol pyrophosphate toxicity, which led us to the discovery that *asp1-H397A aps1*∆ *tgp1*∆ was suppressed by *nab2*∆. Studies of Nab2 in budding yeast implicate this nuclear poly(A) binding protein in mRNA transport (via its physical interaction with the nuclear pore complex) and in mRNA quality control (39). In addition, it was reported that depletion of Nab2 in budding yeast elicited a defect in 3'-processing and termination of nascent RNAs, resulting in transcriptional readthrough into downstream transcription units (45). Such an effect of Nab2 deletion, if it applies in fission yeast, would potentially account for *nab2*∆ reversal of the precocious 3'-processing/termination that underpins inositol pyrophosphate toxicity. Further studies aimed at defining the nexus of Nab2 with 3'-processing/termination, nuclear export, and inositol pyrophosphate signaling in fission yeast are warranted.

## MATERIALS AND METHODS

### Fission yeast strains

A list of strains used in this study is provided in Table S2.

### Spot tests of fission yeast growth

Cultures of *S. pombe* strains were grown in liquid ePMGT (enhanced pombe minimal glutamate with thiamine) (25) or YES (yeast extract with supplements) (46) medium until *A*_600_ reached 0.3–0.8. The cultures were adjusted to an *A*_600_ of 0.1, and aliquots (3 µL) of serial fivefold dilutions were spotted to ePMGT or YES agar. The plates were photographed after incubation for 2 days at 34°C, 2 to 2.5 days at 30°C and 37°C, 4 days at 25°C, and 6 days at 20°C.

### Preparation of cell extracts and enrichment for inositol polyphosphates

Cultures of *S. pombe* strains (three biological replicates for each strain) were grown in liquid ePMGT medium at 30°C. Aliquots corresponding to 10 *A*_600_ units (2–3 × 10^8^ cells) of exponentially growing cells (*A*_600_ between 0.6 and 0.8) were harvested by centrifugation. The cells were washed in ice-cold water and resuspended in 1 mL of 1 M perchloric acid. After snap-freezing in liquid nitrogen, the samples were stored at −80°C. The samples were thawed, mixed briefly by vortexing, and cell debris was removed by centrifugation at 16,200 × *g* for 5 minutes at 4°C. Acid-extracted inositol polyphosphates in the supernatants were then purified using titanium dioxide beads (GL Sciences 5020-75000) as described by Wilson and Saiardi (32). In brief, the cell supernatants were mixed with TiO_2_ beads (4 mg per sample) that had been equilibrated in 1 M perchloric acid and incubated on a nutator for 20 min at 4°C. The TiO_2_ beads (plus bound material) were recovered by centrifugation (5,000 × *g* for 1 minute at 4°C) and washed twice with 1 M perchloric acid. Inositol polyphosphates were eluted from the beads in two cycles of resuspension in 200 µL of 3% ammonium hydroxide, rotation of the samples for 5 minutes at 4°C, and centrifugation (5,000 × *g* for 1 minute). The combined eluates (400 µL per sample) were evaporated to dryness in a vacuum centrifuge (Savant SpeedVac) at 42°C for ~3 hours. The dried samples were stored at −80°C and resuspended in 30 µL of water immediately prior to analysis by CE-ESI-MS.

### Capillary electrophoresis electrospray ionization mass spectrometry (CE-ESI-MS)

The analyses were conducted as described (29–31). Inositol polyphosphate concentrations in the TiO_2_-enriched cell extracts were determined with an Agilent 7100 capillary electrophoresis (CE) system coupled to a triple quadrupole tandem mass spectrometry Agilent 6495 c system. Ionization was performed with the help of an Agilent Jet Stream (AJS) electrospray ionization (ESI) source and a liquid coaxial interface from Agilent. Ionization spray was stabilized with sheath liquid containing a water–isopropanol (1:1) mixture, pumped with an Agilent 1200 isocratic LC pump and a 1:100 splitter to a flow rate of 10 µL/minute. Cell extract samples were spiked with heavy (^13^C_6_) inositol polyphosphate standards (47, 48; provided by Dorothea Fiedler), and 20 nL aliquots of the samples were injected by applying pressure of 100 mbar for 20 seconds. A bare fused silica capillary (length 100 cm; inner diameter 50 µm) was used for measurements. Ammonium acetate (35 mM, adjusted to pH 9.75 with ammonia solution) was used as the background electrolyte (BGE). By applying a voltage of +30 kV, a stable CE current of 22 µA was received. The gas temperature of the Nebulizer was set to 150°C with a flow rate of 11 L/minute and a pressure of 8 psi. Sheath gas flow was set at 8 L/minute with a temperature of 175°C. Capillary voltage was set to 2,000 V and nozzle voltage to 2,000 V. The negative high-pressure radio frequency was 70 V, and the low-pressure radio frequency was 40 V. The multiple reaction monitoring (MRM) transitions were optimized with the MassHunter Optimizer software.

The levels of 1,5-IP_8_, 1-IP_7_, 5-IP_7_, and IP_6_ are reported in bar graph format in the figures as total pmol in the 30 µL sample of the TiO_2_-enriched cell extract (three biological replicates ±SEM). The *P* values for differences in levels between wild-type and mutant fission yeast strains were determined via Welch’s *t*-test implemented in Prism. The inositol pyrophosphate and IP_6_ levels reported in Fig. 2 for an ensemble of wild-type and mutant strains are derived from a single contemporaneous experiment. The levels reported in Fig. 3 and 4 for wild-type cells and a different set of mutants are derived from a separate experiment performed at a later date. Whereas the wild-type level of IP_6_ is several-fold higher in the second experiment, the wild-type levels of 1,5-IP_8_, 1-IP_7_, and 5-IP_7_ are comparable between experiments.

### Cell-surface acid phosphatase activity

Cells were grown at 30°C in liquid ePMGT or YES medium. Aliquots of exponentially growing cultures were harvested, washed, and resuspended in water. To quantify acid phosphatase activity, reaction mixtures (200 µL) containing 100 mM sodium acetate (pH 4.2), 10 mM *p*-nitrophenylphosphate, and cells (ranging from 0.01 to 0.1 *A*_600_ units) were incubated for 5 minutes at 30°C. The reactions were quenched by addition of 1 mL of 1 M sodium carbonate, the cells were removed by centrifugation, and the absorbance of the supernatant at 410 nm was measured. Acid phosphatase activity is expressed as the ratio of *A*_410_ (*p*-nitrophenol production) to *A*_600_ (cells). The data are averages (±SEM) of at least three assays using cells from at least three independent cultures.

### Whole-genome sequencing

After PicoGreen quantification and quality control by Agilent BioAnalyzer, 500 ng aliquots of genomic DNA were sheared using a LE220-plus Focused-ultrasonicator (Covaris catalog # 500569). Sequencing libraries were prepared using the KAPA Hyper Prep Kit (Kapa Biosystems KK8504) with modifications. DNA libraries were subjected to size selection by mixture with 0.5 vol of aMPure XP beads (Beckman Coulter catalog # A63882) after post-ligation cleanup. Libraries were not amplified by the PCR and were pooled equivolume for sequencing. Samples were run on a NovaSeq 6000 in a 150 bp/150 bp paired-end run using the NovaSeq 6000 SBS v1 Kit and an S1 flow cell (Illumina). The average number of read pairs per sample was 8.7 million. The average coverage of the genome was 206-fold (range 26-fold to 360-fold).

### Mapping suppressor mutations

The FASTA file for the *S. pombe* genome was accessed from Pombase. The whole-genome sequencing data from the parental *Hat* strain (*asp1-H397A aps1*∆ *tgp1*∆) and the *Hat-S* suppressor mutants were aligned to the genome using Bowtie2 (49). The resulting SAM files were converted to BAM files using Samtools (50). Variants were identified by BCFtools (51) using the criteria of adjusted mapping quality = 40, minimum base quality = 20, and disabled probabilistic realignment for the computation of base alignment quality (BAQ) for considering variations or insertion–deletion events. The multi-allelic caller protocol was used for variant calling in BCFtools. Variants were annotated using SnpEff, with its in-built genome version for *S. pombe* (52). Variants were further filtered by removing all variations with an average mapping quality ≤25 (Phred scale). All variants present in the parental strain were excluded as non-causal mutations.

### Transcriptome profiling by RNA-seq

RNA was isolated from *S. pombe* wild-type and *asp1-H397A aps1*∆ *siw14*∆ *spx1*∆ cells (three biological replicate cultures of each strain) that were grown in liquid ePMGT medium at 30°C to an *A*_600_ of 0.5 to 0.6. Cells were harvested by centrifugation, and total RNA was extracted via the hot phenol method. The integrity of total RNA was gauged with an Agilent Technologies 2100 Bioanalyzer. The NEB Ultra II Directional RNA library Prep plus Poly A isolation module kit was used to prepare the libraries for paired-end sequencing using a NovaSeq X-Plus system. FASTQ files bearing paired-end reads of length 51 bases (total paired reads of 20 million to 26.4 million per biological replicate) were mapped to the *S. pombe* genome (ASM294v2.28) using HISAT2-2.1.0 with default parameters (53). In the data sets, 94–96% of the reads were mapped to unique genomic loci. The resulting SAM files were converted to BAM files using Samtools (50). Count files for individual biological replicates were generated with HTSeq-0.10.0 (54) using exon annotations from Pombase (GFF annotations, genome-version ASM294v2; source “ensembl”). RPKM analysis and pairwise correlations were performed as described previously (55). Read densities (RPKM) for individual genes were highly reproducible between biological replicates (Pearson coefficients of 0.971 to 0.985). Differential gene expression and fold change analysis were performed in DESeq2 (56). The cutoff for further evaluation was set for genes with an adjusted *P* value (Benjamini–Hochberg corrected) of ≤0.05 and were up or down by at least twofold in *asp1-H397A aps1*∆ *siw14*∆ *spx1*∆ versus wild-type. Genes were further filtered on the following criteria: (i) genes that were ≥2-fold up and the average normalized read count for the mutant strain were ≥100; and (ii) genes that were ≥2-fold down and the average normalized read count for the wild-type strain were ≥100.

### *S. pombe nab2*∆ strain

PCR amplification and standard cloning methods were employed to construct a plasmid in which a *kanMX* drug-resistance cassette is flanked by the 751 bp and 727 bp genomic DNA segments upstream and downstream of the *nab2* ORF, thereby deleting *nab2* from nucleotides + 4 to +912 (relative to the translation start codon +1). The *nab2*∆*::kanMX* disruption cassette was excised from the plasmid and transfected into diploid *S. pombe* cells. G418-resistant transformants were selected and analyzed by Southern blotting to confirm correct integration at the *nab2* locus. Antibiotic-resistant *nab2*∆ haploids were isolated after sporulation of the heterozygous diploids.

## Data Availability

The RNA-seq data in this publication have been deposited in NCBI’s Gene Expression Omnibus and are accessible through GEO Series accession number GSE285739.

## SUPPLEMENTAL MATERIAL

Tables S1 and S2 mbio.00389-25-s0001.pdf RNA-seq and *S. pombe* strain list.

ASM does not own the copyrights to Supplemental Material that may be linked to, or accessed through, an article. The authors have granted ASM a non-exclusive, world-wide license to publish the Supplemental Material files. Please contact the corresponding author directly for reuse.
