# Supplementary material for: Tandem inactivation of inositol pyrophosphatases Asp1, Siw14, and Aps1 illuminates functional redundancies in inositol pyrophosphate catabolism in fission yeast
Source: mBio. 2025 Apr 16;16(5):e00389-25. doi: 10.1128/mbio.00389-25 (PMC12077094; doi:10.1128/mbio.00389-25)
Supplement: Tables S1 and S2 — RNA-seq and S. pombe strain list. [file mbio.00389-25-s0001.pdf]

Supplemental Material

Tandem inactivation of inositol pyrophosphatases Asp1, Siw14, and Aps1 illuminates functional redundancies in inositol pyrophosphate catabolism in fission yeast

Beate Schwer, Isabel Prucker, Ana M. Sanchez, Jill Babor, Henning J. Jessen,

and Stewart Shuman

Supplemental Table S1. Lists of protein-coding transcripts that are downregulated or upregulated by ≥2-fold (expressed as log2 Fold Change) in *aps1-H397A siw14*Δ *asp1*Δ *tgp1*Δ cells vis-à-vis wild-type cells.

Supplemental Table S2. List of *S. pombe* strains used in this study.

Systematic ID	Gene name	Product	log2FoldChange
SPBC359.02	alr2	alanine racemase Alr2	-4.26
SPAPB8E5.05	mfm1	M-factor precursor Mfm1	-3.78
SPBPB21E7.04c	cmt2	O-methyltransferase, human COMT catechol homolog 2	-3.27
SPBC359.01		amino acid transmembrane transporter	-2.88
SPBP26C9.02c	car1	arginase Car1	-2.73
SPAC1039.09	isp5	amino acid transmembrane transporter Isp5	-2.66
SPBPB10D8.02c		arylsulfatase	-2.65
SPBPB2B2.01	nrg1	amino acid transmembrane transporter Nrg1	-2.54
SPAC11D3.06		MatE family transmembrane transporter	-2.40
SPBC839.06	cta3	P-type ATPase, potassium exporting Cta3	-2.39
SPAPB24D3.03	agm2	agmatinase 2, Agm2	-2.30
SPAC513.03	mfm2	M-factor precursor Mfm2	-2.21
SPBC1683.06c	urh1	uridine ribohydrolase Urh1	-2.15
SPBC1683.08	ght4	plasma membrane hexose:proton symporter, unknown specificity Ght4	-2.09
SPAC27D7.03c	mei2	RNA-binding protein involved in meiosis Mei2	-2.04
SPAC11H11.04	mam2	pheromone p-factor receptor Mam2	-2.02
SPAC11E3.06	map1	DNA-binding transcription factor, MADS-box Map1	-1.97
SPAC11D3.03c		aminomethyltransferase-like and DUF1989 family protein, implicated in urea metabolism	-1.94
SPAC977.13c		hydrolase, pseudogene (4 frameshifts)	-1.89
SPBC8E4.03	agm3	agmatinase 3, Agm3	-1.81
SPBPB2B2.02	say1	ER sterol deacetylase Say1	-1.78
SPAC1399.04c	uck2	uracil phosphoribosyltransferase Uck2	-1.77
SPBC359.04c	pfl7	cell surface glycoprotein, flocculin Pfl7, DIPSY family	-1.66
SPAC1002.17c	urg2	uracil phosphoribosyltransferase	-1.64
SPAC1002.19	urg1	GTP cyclohydrolase II Urg1	-1.62
SPCC1223.09	uro1	uricase Uro1	-1.62
SPBC32C12.02	ste11	DNA-binding transcription factor Ste11	-1.61
SPCC548.07c	ght1	plasma membrane high-affinity glucose:proton symporter Ght1	-1.60
SPAP11E10.01		ornithine cyclodeaminase-like protein	-1.58
SPCC737.04		UPF0300 family protein 6	-1.56
SPAPJ695.02		Schizosaccharomyces pombe specific protein	-1.54
SPAC977.15		dienelactone hydrolase family, implicated in cellular detoxification	-1.54
SPAC1556.04c	cdd1	cytidine deaminase Ccd1	-1.52
SPAC1399.06		dubious	-1.51
SPAC1565.04c	ste4	MAPK cascade adaptor protein Ste4	-1.49
SPAC56F8.15		Schizosaccharomyces pombe specific protein	-1.48
SPAC1F8.01	ght3	plasma membrane gluconate:proton symporter Ght3	-1.48
SPAC922.07c	atd2	aldehyde dehydrogenase	-1.48
SPAC869.05c	sul2	plasma membrane sulfate transmembrane transporter Sul2	-1.47
SPAC31G5.09c	spk1	MAP kinase Spk1	-1.44
SPBC359.03c	aat1	plasma membrane amino acid transmembrane transporter Aat1	-1.43
SPCC569.07		aromatic aminotransferase	-1.43
SPAC1039.01		amino acid transmembrane transporter	-1.42
SPCC338.12	pbi2	vaculoar proteinase B inhibitor Pbi2	-1.39
SPAC11D3.19		Schizosaccharomyces pombe specific protein	-1.37
SPBC16A3.13	meu7	alpha-amylase homolog Aah4	-1.36
SPAC664.13		Schizosaccharomyces pombe specific protein	-1.36
SPBPB2B2.05		class I glutamine amidotransferase family protein	-1.34
SPAC1399.05c	toe1	DNA-binding transcription factor, zf-fungal binuclear cluster type	-1.33
SPAC977.16c	dak2	dihydroxyacetone kinase Dak2	-1.32
SPBPB10D8.01		cysteine transmembrane transporter	-1.32
SPAC1039.10	mmf2	mitochondrial matrix protein, YjgF family protein Mmf2, reactive intermediate imine deaminase A homolog	-1.31
SPCC794.02	wtf5	wtf antidote-like meiotic drive suppressor Wtf5	-1.29
SPCC417.02	dad5	DASH complex subunit Dad5	-1.26
SPAC1002.18	urg3	DUF1688 family fungal conserved protein, implicated in uracil or riboflavin metabolism	-1.24
SPBC725.03		pyridoxamine 5'-phosphate oxidase	-1.24
SPAP7G5.06	per1	plasma membrane amino acid transmembrane transporter Per1	-1.22
SPAC521.03		short chain dehydrogenase, human DHRS7 family	-1.21
SPAC1F8.05	isp3	spore wall structural constituent Isp3	-1.19
SPBC36.02c		spermidine family transmembrane transporter	-1.17
SPAC869.10c	put4	plasma membrane proline transmembrane transporter Put4	-1.17
SPBPB21E7.07	aes1	phenazine biosynthesis PhzF protein family	-1.15
SPBPB21E7.08		pseudogene	-1.14
SPBC1683.12		carboxylic acid transmembrane transporter	-1.12
SPBC365.12c	ish1	nuclear envelope Ish domain protein Ish1	-1.11
SPAC23H3.15c	ddr48	DNA damage-responsive protein Ddr48	-1.11
SPAC110.05	SPAC110.06	dubious	-1.10
SPAC167.06c	mug143	Schizosaccharomyces specific protein Mug143	-1.09
SPAC30D11.01c	gto2	extracellular alpha-glucosidase Gto2	-1.06
SPBC23G7.13c		plasma membrane urea transmembrane transporter	-1.04
SPBC21C3.19	rtc3	SBDS family protein Rtc3	-1.01

Systematic ID	Gene name	Product	log2FoldChange
SPBCPT2R1.08c	tlh2	RecQ type DNA helicase Tlh1	2.78
SPAC27D7.09c		But2 family protein, similar to cell surface molecules	2.26
SPAC186.05c	gdt1	Golgi calcium and manganese antiporter Gdt1	2.07
SPBC947.04	pfl3	cell surface glycoprotein, flocculin Pfl3, DIPSY family	1.75
SPCC191.11	inv1	external invertase, beta-fructofuranosidase Inv1	1.67
SPBC530.02		transmembrane transporter	1.29
SPBC1711.15c		Schizosaccharomyces pombe specific protein	1.25
SPCC622.19	jmj4	histone demethylase Jmj4	1.17
SPAC23D3.05c		alcohol dehydrogenase pseudogene	1.07
SPAC1B3.16c	vht1	plasma membrane vitamin H transmembrane transporter Vht1	1.06
SPAC4H3.08	fox2	3-hydroxyacyl-CoA dehydrogenase Fox2	1.05
SPBC409.08		spermine family transmembrane transporter	1.05
SPCC364.06	nap1	histone H2A-H2B chaperone Nap1	1.02

Table S2. List of *S. pombe* strains used in this study.

Strain	Genotype
AS612	*h+ ssu72-C13S::natMX*
AS2308	*h- asp1-H397A::kanMX aps1Δ::hygMX ssu72-C13S::natMX*
BS54	*h- spx1Δ::kanMX*
BS400	*h+ asp1-H397A::natMX spx1Δ::kanMX aps1Δ::hygMX*
BS828	*h- aps1Δ::hygMX spx1Δ::kanMX siw14Δ::natMX*
AGP237	*h- tgp1Δ::ura4*$^{+}$
BS957	*h+ tgp1Δ::ura4*$^{+}$ *aps1Δ::hygMX*
BS958	*h+ tgp1Δ::ura4*$^{+}$ *siw14Δ::hygMX*
BS971	*h+ tgp1Δ::ura4*$^{+}$ *aps1Δ::kanMX siw14Δ::hygMX*
BS1011	*h+ asp1-H397A::natMX aps1Δ::hygMX tgp1Δ::ura4*$^{+}$ *[Hat]*
BS1452	*h+ siw14Δ::hygMX asp1-H397A::natMX aps1Δ::kanMX spx1Δ::ura4MX*
BS1454	*h- asp1-H397A::natMX aps1Δ::hygMX spx1-Y26A-K30A-K34A::kanMX*
BS1456	*h- asp1-H397A::natMX aps1Δ::hygMX spx1-C374A-C377A::kanMX*
BS1402	*h- asp1-H397A::natMX aps1Δ::hygMX tgp1Δ::ura4*$^{+}$ *nab2-R192L kcs1-Q739K [Hat-S1]*
BS1403	*h+ asp1-H397A::natMX aps1Δ::hygMX tgp1Δ::ura4*$^{+}$ *kcs1-Q739K [Hat-S2]*
BS1404	*h- asp1-H397A::natMX aps1Δ::hygMX tgp1Δ::ura4*$^{+}$ *kcs1-R738T [Hat-S3]*
BS1405	*h+ asp1-H397A::natMX aps1Δ::hygMX tgp1Δ::ura4*$^{+}$ *spx1-F393V [Hat-S4]*
BS1406	*h- asp1-H397A::natMX aps1Δ::hygMX tgp1Δ::ura4*$^{+}$ *kcs1-G765R [Hat-S5]*
BS1407	*h+ asp1-H397A::natMX aps1Δ::hygMX tgp1Δ::ura4*$^{+}$ *kcs1-Q739K [Hat-S6]*
BS1408	*h- asp1-H397A::natMX aps1Δ::hygMX tgp1Δ::ura4*$^{+}$ *spx1-(298fs) [Hat-S7]*
BS1409	*h+ asp1-H397A::natMX aps1Δ::hygMX tgp1Δ::ura4*$^{+}$ *swd22-S125R [Hat-S8]*
BS1505	*h+ siw14Δ::kanMX aps1Δ::hygMX asp1Δ::kanMX*
BS1509	*h+ siw14-C189S::hygMX aps1-E89A-E93A::kanMX spx1Δ::ura4MX*
BS1511	*h+ siw14-C189S::hygMX aps1-E89A-E93A::kanMX tgp1Δ::ura4+*
BS1513	*h+ asp1-H397A::natMX siw14-C189S::hygMX aps1-E89A-E93A::kanMX spx1Δ::ura4MX*
ASY236	*h- nab2Δ::kanMX*
JTB1047	*h+ asp1-H397A::natMX aps1Δ::hygMX tgp1Δ::ura4+ nab2Δ::kanMX*

The strains are *leu1-32 ura4-D18 his3-D1* and either *ade6-m216 or ade6-m210*. Mating types were determined by mixing cells with each of the parental *h+* and *h-* strains on malt agar and observing tetrads after 24-48 h of incubation. Abbreviations of the strain names used in the text and figures text are provided in [ ] brackets.
